# ADDIS‐Graphs for Online Error Control With Application to Platform Trials

**DOI:** 10.1002/bimj.70075

**Published:** 2025-09-28

**Authors:** Lasse Fischer, Marta Bofill Roig, Werner Brannath

**Affiliations:** ^1^ Competence Center for Clinical Trials Bremen University of Bremen Bremen Germany; ^2^ Department of Statistics and Operations Research Universitat Politècnica de Catalunya – BarcelonaTech Barcelona Spain

**Keywords:** false discovery rate, familywise error rate, graphical testing procedures, online multiple testing, platform trials

## Abstract

In contemporary research, online error control is often required, where an error criterion, such as familywise error rate (FWER) or false discovery rate (FDR), shall remain under control while testing an a priori unbounded sequence of hypotheses. The existing online literature mainly considered large‐scale studies and constructed powerful but rigid algorithms for these. However, smaller studies, such as platform trials, require high flexibility and easy interpretability to take study objectives into account and facilitate the communication. Another challenge in platform trials is that due to the shared control arm some of the p‐values are dependent and significance levels need to be prespecified before the decisions for all the past treatments are available. We propose adaptive‐discarding‐Graphs (ADDIS‐Graphs) with FWER control that due to their graphical structure perfectly adapt to such settings and provably uniformly improve the state‐of‐the‐art method. We introduce several extensions of these ADDIS‐Graphs, including the incorporation of information about the joint distribution of the p‐values and a version for FDR control.

## 1 Introduction

In classical multiple testing m∈N, null hypotheses $H_{1},\text{…},H_{m}$ are prespecified at the beginning of the evaluation. Modern data analysis, however, requires dynamic and flexible decision making. This led to the establishment of online multiple testing, where a potentially infinite stream of hypotheses ${\left(H_{i}\right)}_{i\in N}$ is tested sequentially (Foster and Stine 2008; Javanmard and Montanari 2018). This means, at each step i∈N, a decision is made on the current hypothesis $H_{i}$ while having access only to the previous hypotheses and decisions. Since the number of future hypotheses is unknown as well, it is usually assumed to be infinite. Such online multiple testing problems can be found in many different research areas. Examples are platform trials (Robertson et al. 2023a; Zehetmayer et al. 2022), sequential modifications of machine learning algorithms (Feng et al. 2021, 2022), growing data repositories (Aharoni and Rosset 2014; Robertson et al. 2023b), and continuous A/B testing in the tech industry (Kohavi et al. 2013; Ramdas et al. 2017).

A widely known multiplicity adjustment in classical multiple testing is the Bonferroni correction, where an individual hypothesis $H_{i}$ is rejected if its corresponding p‐value $P_{i}$ is less than or equal to α/m. Bonferroni's inequality immediately implies that the adjustment provides familywise error rate (FWER) control, where FWER is defined as the probability of rejecting any true null hypothesis and by controlling we mean that the FWER is bounded by α. In a seminal paper, Holm (1979) showed that the individual significance levels α/m can even be increased if some of the hypotheses are rejected, without violating FWER control. However, classical multiple testing procedures cannot be applied simply to online multiple testing. The two previously mentioned procedures illustrate the difficulty of online multiple testing. First, the number of hypotheses m in online testing is not prespecified in advance and could even be infinite. Second, data information about the other hypotheses can improve the multiple testing procedure, but in online multiple testing the individual significance level $\alpha_{i}$ for a hypothesis $H_{i}$ can only depend on the *previous* p‐values $P_{1},\text{…},P_{i-1}$.

While Bonferroni‐like adjustments can be transferred to online multiple testing (Foster and Stine 2008), they usually lead to low power (Tian and Ramdas 2021). For this reason, Tian and Ramdas (2021) have established the following condition that can be used to prove FWER control for adaptive discarding (ADDIS) online multiple testing procedures:

(1) $$\begin{array}{c} \sum_{i=1}^{\infty }\frac{\alpha_{i}}{\tau_{i}-\lambda_{i}}1\left\{\lambda_{i}<P_{i}\leq \tau_{i}\right\}\leq \alpha , \end{array}$$

where $\alpha_{i}\in \left(0,1\right)$ is the individual significance level of $H_{i}$, $\tau_{i}\in \left(\alpha_{i},1\right]$, $\lambda_{i}\in \left[0,\tau_{i}\right)$ are hyperparameters and 1{·} denotes the indicator function. The condition (1) can be interpreted as having a total level of α that can be spend among all hypotheses. If $\lambda_{i}<P_{i}\leq \tau_{i}$, then we lose $\alpha_{i}/\left(\tau_{i}-\lambda_{i}\right)$ of this level. But if $P_{i}\leq \lambda_{i}$ or $P_{i}>\tau_{i}$, then we do not lose any significance level, meaning $\alpha_{i}$ can be reused for future hypotheses. The idea is that p‐values corresponding to true hypotheses are often conservative and, therefore, tend to be large, and p‐values corresponding to false hypotheses tend to be small such that many significance levels can be reused in the future. While (1) is helpful in showing that a given procedure controls the FWER, it is nonconstructive and thereby of only little help for the construction of ADDIS procedures.

As shown by Tian and Ramdas (2021), $\alpha_{i}$, $\tau_{i}$, and $\lambda_{i}$ are only allowed to depend on information that is independent of $P_{i}$ in order to deduce FWER control from condition (1). The problem is that to exploit (1), $\alpha_{i}$ needs to incorporate information about the previous indicators $1\{\lambda_{j}<P_{j}\leq \tau_{j}\}$, j<i, and thus of the p‐value $P_{j}$. To see this, note that if $\alpha_{i}$ does not use information about the previous p‐values, we can only guarantee that (1) is fulfilled by choosing $\alpha_{i}$, i∈N, such that $\sum_{i=1}^{\infty }\alpha_{i}\leq \alpha$ (and setting $\tau_{i}=1$ and $\lambda_{i}=0$ to make the sum as small as possible), which is equivalent to the conservative online Bonferroni (Tian and Ramdas 2021; Foster and Stine 2008). Therefore, to construct powerful procedures via (1), either all or at least some p‐values need to be independent, as this allows to choose $\alpha_{i}$ based on the previous p‐values that are independent of $P_{i}$. Another issue that may limit the choice of individual significance levels is when the hypotheses are tested in an asynchronous manner (Zrnic et al. 2021). That means an individual significance level $\alpha_{i}$ needs to be determined before the information $1\{\lambda_{j}<P_{j}\leq \tau_{j}\}$ is available for all previous p‐values $P_{j}$, j<i. In general, for each hypothesis $H_{i}$ one can construct a conflict set (Zrnic et al. 2021) that specifies on which of the previous p‐values $\alpha_{i}$ cannot depend (e.g., due to asynchrony) or must not depend (e.g., due to dependence), as otherwise it would violate the FWER control.

For example, in a platform trial many treatment arms $T_{1},T_{2},\text{…}$ are compared to the same control group. However, in contrast to multiarm trials, not all treatment arms are in the platform from the beginning but can be added or dropped at any time and the total number of treatment arms is unknown (Saville and Berry 2016) (see panel A of Figure 2 for an illustration). Hence, this can be interpreted as an online multiple testing problem (Robertson et al. 2023a). Throughout the paper, we assume that only concurrent controls are used, meaning for the evaluation of a treatment arm only control data of those patients is included that were randomized while the corresponding treatment arm was in the platform. This yields a local dependence structure of the p‐values (Zrnic et al. 2021), since overlapping treatment arms share some control data, while p‐values for nonoverlapping treatment arms are independent. Furthermore, the individual significance level for a hypothesis sometimes needs to be determined when the treatment arm enters the platform in order to, for instance, make sample size considerations, while the test decision is obtained when the treatment arm leaves the platform. In this case, overlapping treatment arms also conflict due to an asynchronous testing process.

**FIGURE 1:**
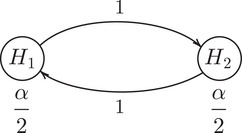
The Bonferroni–Holm correction (Holm 1979) represented as a graphical procedure (Bretz et al. 2009).

**FIGURE 2:**
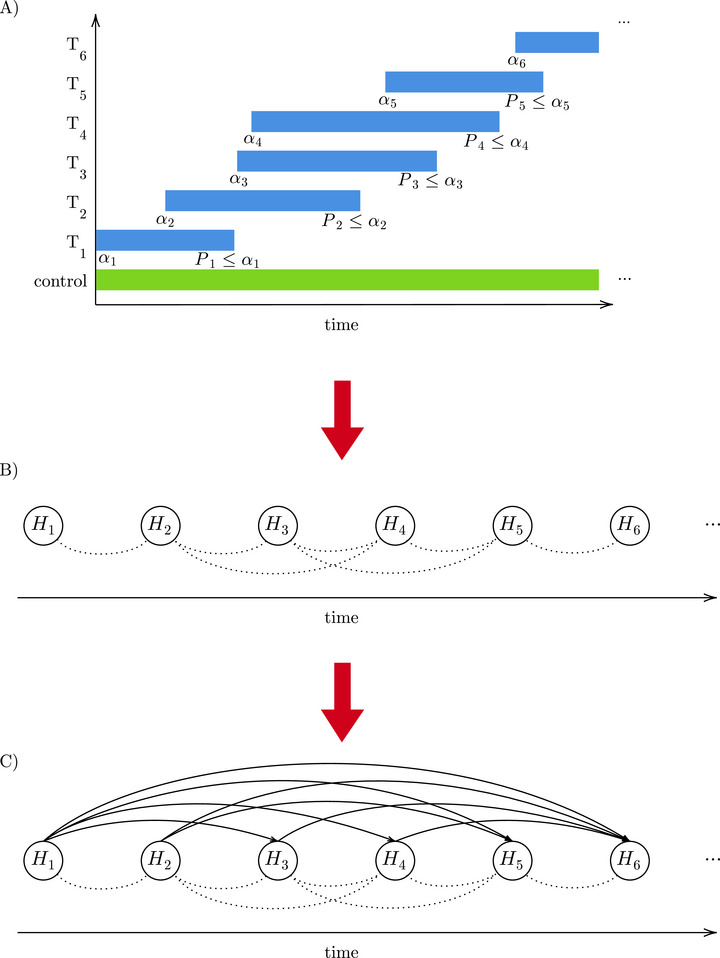
Transferring a platform trial into a graphical procedure. Panel A shows an illustration of a (toy) platform trial. Overlapping treatment arms create conflicts between the corresponding hypotheses due to shared control data and asynchronous testing, where conflict means that the significance level of one hypothesis is not allowed to depend on the p‐value of another. In panel B, the platform trial is transferred into a graphical structure where the hypotheses are represented by nodes and conflicting hypotheses (overlapping treatment arms) are connected by dotted lines. In panel C, it is shown how a graphical procedure, like the ADDIS‐Graph proposed in this paper, can easily adapt to these conflict sets by distributing significance level only between nonconflicting hypotheses.

For trials with multiple study objectives, Bretz et al. (2009) proposed a graphical approach for FWER control to handle multiple test procedures in the classical multiple testing setting. In a graphical procedure, the hypotheses are represented by nodes which are connected by weighted vertices that illustrate the level allocation in case of a rejection. For example, the Bonferroni–Holm correction (Holm 1979) for two hypotheses is illustrated in Figure 1. Initially, both hypotheses are tested at level α/2. However, if one of the hypotheses is rejected, its level can be distributed to the remaining hypothesis such that it is tested at level α. This graphical representation has advantages like facilitating the illustration of study objectives and prioritization of hypotheses. Robertson et al. (2020) extended this graphical approach to other error rates than FWER.

Tian and Ramdas (2021) have introduced the ADDIS‐Spending as a concrete algorithm satisfying the condition (1). However, this algorithm does not exploit the full potential of the condition and is particularly inefficient under conflict sets. For this reason, in this paper, we propose the ADDIS‐Graph, which is a constructive procedure that encompasses all possible online procedures satisfying condition (1) and uniformly improves the ADDIS‐Spending under conflict sets. Furthermore, it is a graphical procedure (Bretz et al. 2009), which is particularly useful for complex trial designs such as those needed in platform trials. For example, consider panel B of Figure 2, where the platform trial is transferred into a graphical structure. The dotted lines represent the conflicts between the hypotheses due to the local dependency/asynchrony caused by overlapping treatment arms. Due to its high flexibility, a graphical multiple testing procedure can easily adapt to this structure by distributing the significance level only between hypotheses that are not connected. For example, the level of hypothesis $H_{1}$ may be distributed to hypotheses $H_{3}$, $H_{4}$, $H_{5}$, $H_{6}$ and onward but not to hypothesis $H_{2}$, and the level of hypothesis $H_{2}$ may be distributed to $H_{5}$, $H_{6}$ and onward but not to $H_{3}$ and $H_{4}$ (see panel C of Figure 2).

In platform trials, there has been a discussion about which error rate is to be controlled. Some argue that in such clinical trials, strict control of FWER should be used and might also be a regulatory requirement (Robertson et al. 2023a; Wason et al. 2014); while others recommend controlling weaker error criteria, such as the false discovery rate (FDR), to avoid an increase of type II errors (Robertson et al. 2023a; Zehetmayer et al. 2022). The purpose of this paper is not to discuss which error rate is most appropriate but to construct multiple testing procedures that are powerful and easy to interpret in such complex online settings. We focus on FWER control and, in addition, discuss extensions of our methods to other error rates in Section 6.

### 1.1 Overview of the Paper

We begin with a formal definition of the problem setting (Section 2). In Section 3, we derive the ADDIS‐Graph when no conflicts are present and show that it contains all other online procedures satisfying condition (1). In Section 4, we adapt the ADDIS‐Graph to conflict sets and prove that this leads to a uniform improvement over the ADDIS‐Spending under local dependence in Section 5. Afterward, we consider extensions of the ADDIS‐Graph to other error rates and further improvements (Section 6). Finally, in Sections 7 and 8, we demonstrate the application of the ADDIS‐Graph through a simulation study and application to a real platform trial, respectively. The R‐Code for the simulations and case study is available at the GitHub repository https://github.com/fischer23/Adaptive‐Discard‐Graph. All formal proofs of the theoretical assertions are in the Supporting Information.

## 2 Problem Setting

Let $I_{0}$ be the index set of true hypotheses, R(i) be the index set of rejected hypotheses up to step i∈N, and $V\left(i\right)=I_{0}\cap R\left(i\right)$ denote the index set of falsely rejected hypotheses up to step i. We aim to control the familywise error rate FWER(i)≔P(|V(i)|>0) at each step i∈N, where P denotes the probability under the true configuration of true and false hypotheses. Since FWER(i) is nondecreasing, it is sufficient to control FWER≔P(v>0), where $v≔\lim_{i\to \infty }\left|V\left(i\right)\right|$. The FWER is controlled strongly at level α, if FWER≤α for any configuration of true and false null hypotheses. We assume that each null p‐value $P_{i}$, $i\in I_{0}$, is valid, meaning $P(P_{i}\leq x)\leq x$ for all x∈[0,1]. A hypothesis $H_{i}$ is rejected, if $P_{i}\leq \alpha_{i}$, where $\alpha_{i}\in \left[0,1\right)$ is the individual significance level of $H_{i}$. In order to apply a multiple testing procedure in the online setting, the individual significance levels are only allowed to depend on the previous p‐values. Mathematically, $\alpha_{i}$, i∈N, is measurable with respect to the sigma algebra $G_{i-1}≔\sigma \left(\left\{P_{1},\text{…},P_{i-1}\right\}\right)$.

As in Zrnic et al. (2021), we define $X_{i}\subseteq \left\{1,\text{…},i-1\right\}$ as the index set of previous hypotheses conflicting with $H_{i}$. The conflict set $X_{i}$ includes all indices of previous p‐values that are *not* stochastically independent of $P_{i}$, but can also contain further indices due to asynchrony or other restrictions. It is also assumed that the conflict sets ${\left(X_{i}\right)}_{i\in N}$ are monotone (Zrnic et al. 2021), which means that $j\notin X_{i}$, i>j, implies $j\notin X_{k}$ for all k≥i. This ensures that the information we are allowed to use at each step i∈N is not decreasing over time. For example, this is fulfilled in every platform trial (e.g., Figure 2), since if $T_{j}$ and $T_{i}$, j<i, do not overlap, then $T_{j}$ and $T_{k}$, k≥i, do not overlap as well (if we order the treatments by entry time). Furthermore, each $X_{i}$ can be considered as fixed, although it might depend on information that is independent of $P_{i}$. In case of $X_{i}=\emptyset$ for all i∈N, we speak of trivial conflict sets. In order to conclude FWER control from condition (1), $\alpha_{i}$, $\lambda_{i}$, and $\tau_{i}$ must be measurable with respect to $G_{-X_{i}}≔\sigma \left(\left\{P_{j}:j<i,j\notin X_{i}\right\}\right)$ (Tian and Ramdas 2021). Furthermore, in case of $\tau_{i}<1$, the null p‐values $P_{i}$, $i\in I_{0}$, are required to be uniformly valid, meaning $P(P_{i}\leq xy|P_{i}\leq y)\leq x$ for all x,y∈[0,1]. However, this condition is fulfilled in many of the usual testing problems (Zhao et al. 2019).

## 3 ADDIS‐Graph Under Trivial Conflict Sets

We start with the construction of ADDIS‐Graphs under trivial conflict sets, which means $\alpha_{i}$, $\tau_{i}$, and $\lambda_{i}$ need to be measurable with respect to $G_{i-1}$. Let us first introduce the ADDIS‐Graph formally and then explain its representation as a graphical procedure afterward. For this, we define $U_{i}=1\left\{P_{i}\leq \lambda_{i}\vee P_{i}>\tau_{i}\right\}$.

Definition 3.1 (ADDIS‐Graph under trivial conflict sets) Let ${\left(\gamma_{i}\right)}_{i\in N}$ and ${\left(g_{j,i}\right)}_{i=j+1}^{\infty }$, j∈N, be nonnegative sequences with $\sum_{i\in N}\gamma_{i}\leq 1$ and $\sum_{i=j+1}^{\infty }g_{j,i}\leq 1$ for all j∈N. In addition, let $\tau_{i}\in \left(0,1\right]$ and $\lambda_{i}\in \left[0,\tau_{i}\right)$ be measurable regarding $G_{i-1}$ for all i∈N. The *ADDIS‐Graph* tests each hypothesis $H_{i}$ at significance level

(2) $$\begin{array}{c} \alpha_{i}=\left(\tau_{i}-\lambda_{i}\right)\left(\alpha \gamma_{i}+\sum_{j=1}^{i-1}g_{j,i}U_{j}\frac{\alpha_{j}}{\tau_{j}-\lambda_{j}}\right). \end{array}$$

Theorem 3.2 The ADDIS‐Graph satisfies the condition (1) and thus controls the FWER in the strong sense under the setting in Section 2 when the conflict sets are trivial.

In order to represent this ADDIS‐Graph as a graph, consider ${\tilde{\alpha }}_{i}=\alpha_{i}\frac{1}{\tau_{i}-\lambda_{i}}$ for all i∈N, where $\alpha_{i}$ is the significance level obtained by the ADDIS‐Graph. Equation (2) gives us

(3) $$\begin{array}{c} {\tilde{\alpha }}_{i}=\alpha_{i}\frac{1}{\tau_{i}-\lambda_{i}}=\alpha \gamma_{i}+\sum_{j=1}^{i-1}g_{j,i}U_{j}{\tilde{\alpha }}_{j}. \end{array}$$

**FIGURE 3:**
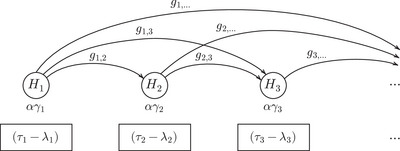
Illustration of the general ADDIS‐Graph. The hypotheses are represented by nodes and the initial level $\alpha \gamma_{i}$ of each $H_{i}$ is provided below the nodes. In case of $P_{j}\leq \lambda_{j}$ or $P_{j}>\tau_{j}$, the current level of $H_{j}$ is distributed to the future hypotheses $H_{i}$, j>i, according to the weights ${\left(g_{j,i}\right)}_{i=j+1}^{\infty }$. Before testing, the current level of $H_{i}$ needs to be multiplied with $\left(\tau_{i}-\lambda_{i}\right)$.

Therefore, $\alpha \gamma_{i}$ can be interpreted as the initial level for $H_{i}$. That means, regardless of the previous p‐values, we always have ${\tilde{\alpha }}_{i}\geq \alpha \gamma_{i}$. Now, this initial level $\alpha \gamma_{i}$ is updated (increased) if previous p‐values $P_{j}$, j<i, lie outside the interval $\left(\lambda_{j},\tau_{j}\right]$. More precisely, if $P_{j}\leq \lambda_{j}$ or $P_{j}>\tau_{j}$, or equivalently, if $U_{j}=1$, we add $g_{j,i}{\tilde{\alpha }}_{j}$ to the individual level for $H_{i}$. Doing this at all previous steps j<i, yields the level ${\tilde{\alpha }}_{i}$ given in (3) for the hypothesis $H_{i}$ at time i. This general approach is illustrated in Figure 3, where the hypotheses are represented by nodes that are connected by weighted vertices indicating the level propagation, like in the classical graphical procedure (Bretz et al. 2009). The rectangles below the nodes clarify that the level ${\tilde{\alpha }}_{i}$ needs to be multiplied with $\left(\tau_{i}-\lambda_{i}\right)$ before comparing it with the p‐value $P_{i}$. Note that this testing factor is only multiplied with the individual significance level when the corresponding hypothesis is tested, but it is not involved in the updating process with the graph.

**FIGURE 4:**
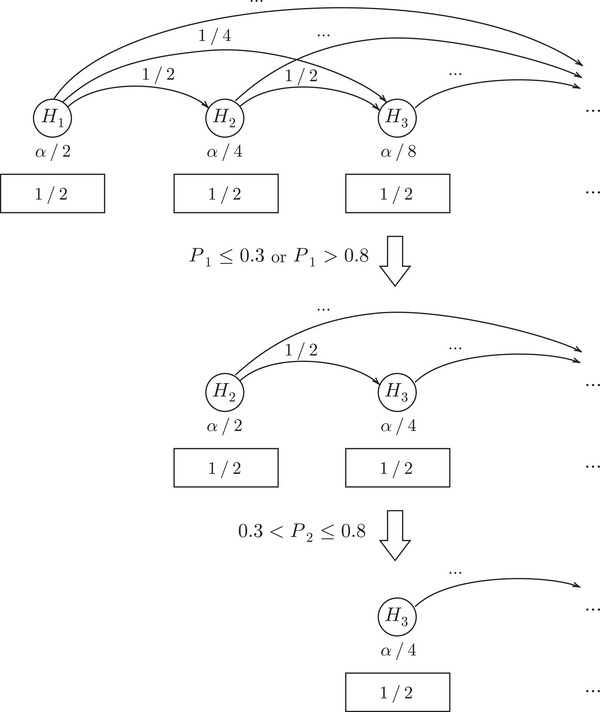
Development of the ADDIS‐Graph described in Example 3.3.

Example 3.3 Suppose $\gamma_{i}={\left(1/2\right)}^{i}$, $g_{j,i}={\left(1/2\right)}^{i-j}$, $\tau_{i}=0.8$, and $\lambda_{i}=0.3$ for all i∈N and j<i. Further suppose $P_{1}\leq 0.3\vee P_{1}>0.8$ and $0.3<P_{2}\leq 0.8$, meaning we can distribute the significance level of the first hypothesis but not of the second. The development of the resulting ADDIS‐Graph is illustrated in Figure 4. Hence, after multiplying with the testing factor 1/2 (see the rectangles below the nodes), we obtain $\alpha_{1}=\alpha_{2}=\alpha /4$ and $\alpha_{3}=\alpha /8$. As we will later see, the ADDIS‐Graph is equivalent to the ADDIS‐Spending by Tian and Ramdas (2021) for this specific choice of weights (see Lemma 5.1), but the ADDIS‐Graph allows to uniformly improve the ADDIS‐Spending if conflicts are present (see Proposition 5.2).

To summarize, in contrast to the classical graphical procedure by Bretz et al. (2009) (see also Figure 1), the ADDIS‐Graph does not distribute the level of $H_{j}$ to other hypotheses in case of a rejection but if $P_{j}\leq \lambda_{j}$ or $P_{j}>\tau_{j}$. The price of this improvement (as we usually choose $\lambda_{j}\geq \alpha_{j}$) is the factor $\left(\tau_{i}-\lambda_{i}\right)$ that needs to be multiplied with the level of $H_{i}$ before testing. Furthermore, the weights ${\left(g_{j,i}\right)}_{j\in N,i>j}$ do not need to be updated, since significance level is only distributed to future hypotheses.

In Definition 3.1, we considered the parameters ${\left(\gamma_{i}\right)}_{i\in N}$ and ${\left(g_{j,i}\right)}_{i=j+1}^{\infty }$, j∈N as fixed. However, $\gamma_{i}$ and $g_{j,i}$ could also be random variables that are measurable regarding $G_{i-1}$, since we only need to ensure that $\alpha_{i}$ is measurable with respect to $G_{i-1}$. With this, the procedures become more flexible. It can even be shown that, in this case, the ADDIS‐Graph is the general ADDIS procedure, thus containing all online procedures satisfying condition (1).

Proposition 3.4 Let $\gamma_{i}$ (i∈N) and $g_{j,i}$ (j∈N, i>j) be measurable with respect to $G_{i-1}$. Then, any online procedure satisfying condition (1) can be written as an ADDIS‐Graph (Definition 3.1).

Note that Proposition 3.4 is not restricted to trivial conflict sets. Thus, if an online procedure ${\left(\alpha_{i}\right)}_{i\in N}$ was adapted to conflict sets ${\left(X_{i}\right)}_{i\in N}$ such that $\alpha_{i}$ is measurable with respect to $G_{-X_{i}}$, it can also be constructed as an ADDIS‐Graph that is given by (2). Therefore, being an ADDIS‐Graph is necessary for an FWER controlling online procedure satisfying condition (1) under any conflict sets. Theorem 3.2 implies that being an ADDIS‐Graph is also sufficient to control the FWER with (1) under trivial conflict sets. In the following section, we introduce a smaller class of ADDIS‐Graphs that are sufficient for FWER control under monotone conflict sets.

## 4 ADDIS‐Graph Under Nontrivial Conflict Sets

**FIGURE 5:**
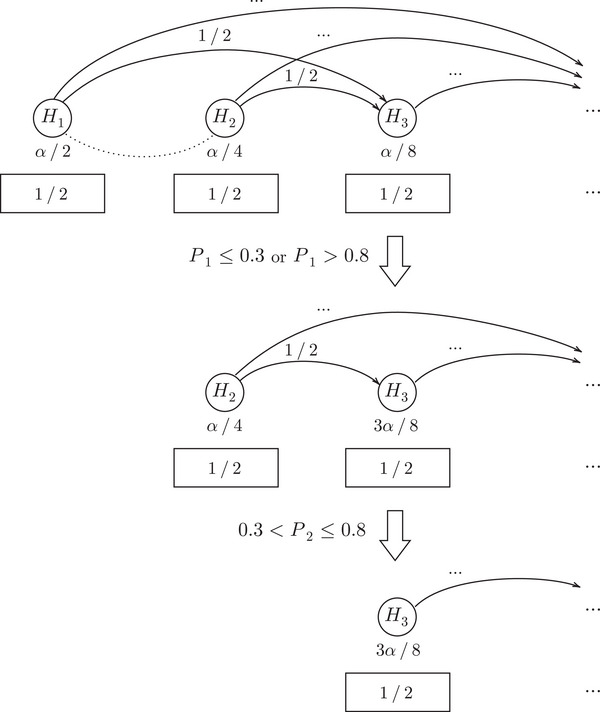
Possible adjustment of the ADDIS‐Graph in Figure 4 to the conflict set $X_{2}=\left\{1\right\}$.

Let us reconsider Example 3.3 (see also Figure 4) but now suppose there is a conflict between $H_{1}$ and $H_{2}$, meaning $X_{2}=\left\{1\right\}$. The ADDIS‐Graph can easily be adjusted to this conflict set by not distributing level between $H_{1}$ and $H_{2}$. We use the ∗ in $g_{j,i}^{*}$ to indicate that the weights are adjusted to conflict sets. Since $g_{1,2}^{*}=0$, we can increase the remaining weights. For example, by setting $g_{1,i}^{*}={\left(1/2\right)}^{i-j-1}$ for all i>3. This is illustrated in Figure 5. Note that due to the conflict between $H_{1}$ and $H_{2}$, the significance level for $H_{2}$ is smaller than in Figure 4, where no conflicts were present. However, the level of $H_{3}$, and all other future hypotheses, increased, such that no significance level is lost overall.

In general, the idea is to receive significance level for hypothesis $H_{i}$ only from hypotheses that are not contained in $X_{i}$.

Definition 4.1 (${\text{ADDIS-Graph}}_{\text{conf}}$) Let the conflict sets be given by ${\left(X_{i}\right)}_{i\in N}$. Let ${\left(\gamma_{i}\right)}_{i\in N}$ be a nonnegative sequence that sums up to 1 and ${\left(g_{j,i}^{*}\right)}_{i=j+1}^{\infty }$ be a nonnegative sequence for all j∈N such that $g_{j,i}^{*}=0$ if $j\in X_{i}$ and $\sum_{i>j,j\notin X_{i}}g_{j,i}^{*}\leq 1$. In addition, let $\gamma_{i}$, $g_{j,i}^{*}$, $\tau_{i}\in \left(0,1\right]$ and $\lambda_{i}\in \left[0,\tau_{i}\right)$ be measurable regarding $G_{-X_{i}}$. The ${\mathrm{ADDIS}-\mathrm{Graph}}_{\mathrm{conf}}$ tests each hypothesis $H_{i}$ at significance level

(4) $$\begin{array}{c} \alpha_{i}=\left(\tau_{i}-\lambda_{i}\right)\left(\alpha \gamma_{i}+\sum_{j=1}^{i-1}g_{j,i}^{*}U_{j}\frac{\alpha_{j}}{\tau_{j}-\lambda_{j}}\right). \end{array}$$

Note that $\alpha_{i}$ from (4) is measurable with respect to $G_{-X_{i}}$, since $g_{j,i}^{*}=0$ for all $j\in X_{i}$ and the conflict sets ${\left(X_{i}\right)}_{i\in N}$ are monotone. With this, the FWER control of ${\text{ADDIS-Graph}}_{\text{conf}}$ comes directly by Theorem 3.2.

Corollary 4.2 The ${\text{ADDIS-Graph}}_{\text{conf}}$ controls the FWER in the strong sense under the setting in Section 2 when conflicts are present.

Also note that for $X_{i}=\emptyset$ for all i∈N the ${\text{ADDIS-Graph}}_{\text{conf}}$ becomes the ADDIS‐Graph under trivial conflict sets (Definition 3.1).

## 5 A Uniform Improvement of the ADDIS‐Spending

The current state‐of‐the‐art method satisfying condition (1) is the ADDIS‐Spending by Tian and Ramdas (2021), which controls the FWER under local dependence. The p‐values ${\left(P_{i}\right)}_{i\in N}$ are said to be locally dependent (Zrnic et al. 2021), if

(5) $$\begin{array}{c} P_{i}⊥P_{i-L_{i}-1},P_{i-L_{i}-2},\text{…},P_{1} \end{array}$$

for some lags ${\left(L_{i}\right)}_{i\in N}$ with $L_{i+1}\leq L_{i}+1$. Zrnic et al. (2021) noted that local dependence is included in the more general concept of conflict sets by defining $X_{i}=\left\{i-1,\text{…},i-L_{i}\right\}$ while the condition $L_{i+1}\leq L_{i}+1$ ensures that these conflict sets are monotone. For example, an intuitive special case of local dependence is batch dependence. That means, there are disjoint groups of p‐values $B_{1}=\left\{P_{1},\text{…},P_{j}\right\}$ for j∈N, $B_{2}=\left\{P_{j},\text{…},P_{k}\right\}$ for k≥j, $B_{3}=\left\{P_{k},\text{…},P_{l}\right\}$ for l≥k, and so on, such that p‐values from the same batch may depend on each other, but hypotheses from different batches are independent. For instance, batch dependence occurs when the data are replaced by fresh and independent data after a period of time.

The ${\mathrm{ADDIS}-\mathrm{Spending}}_{\mathrm{local}}$ is defined as

(6) $$\begin{array}{c} \alpha_{i}^{\text{spend}}=\alpha \left(\tau_{i}-\lambda_{i}\right)\gamma_{t{\left(i\right)}^{\text{loc}}},\;\text{where}\;t{\left(i\right)}^{\text{loc}}=1+L_{i}+\sum_{j=1}^{i-L_{i}-1}\left(1-U_{j}\right), \end{array}$$

where ${\left(\gamma_{i}\right)}_{i\in N}$ is the same as in an ADDIS‐Graph but nonincreasing. Note that $t{\left(i\right)}^{\text{loc}}$ increases when $L_{i}$ increases and thus $\alpha_{i}^{\text{spend}}$ decreases. Therefore, the ${\text{ADDIS-Spending}}_{\text{local}}$ loses significance level due to the dependency of the p‐values. This is the main difference to the ${\text{ADDIS-Graph}}_{\text{conf}}$, where we argued that the same significance level is distributed under any conflict sets, as long as there is a nonconflicting future hypothesis. In the following, we use this to define an ${\text{ADDIS-Graph}}_{\text{conf}}$ that uniformly improves the ${\text{ADDIS-Spending}}_{\text{local}}$. For this, we first show how the ${\text{ADDIS-Spending}}_{\text{local}}$ can be written as something similar to an ADDIS‐Graph.

Lemma 5.1 Let ${\left(\gamma_{i}\right)}_{i\in N}$ be nonincreasing and define

$$\begin{array}{cc} \alpha_{i}^{\text{ind}} & ≔\left(\tau_{i}-\lambda_{i}\right)\left(\alpha \gamma_{i}+\sum_{j=1}^{i-1}g_{j,i}U_{j}\frac{\alpha_{j}^{\text{ind}}}{\tau_{j}-\lambda_{j}}\right)\\ \alpha_{i}^{\text{loc}} & ≔\left(\tau_{i}-\lambda_{i}\right)\left(\alpha \gamma_{i}+\sum_{j=1}^{i-L_{i}-1}g_{j,i}U_{j}\frac{\alpha_{j}^{\text{ind}}}{\tau_{j}-\lambda_{j}}\right). \end{array}$$

Then $\alpha_{i}^{\text{ind}}$ is equivalent to an ADDIS‐Spending under independence ($L_{i}=0\;\forall i\in N$) and $\alpha_{i}^{\text{loc}}=\alpha_{i}^{\text{spend}}$ for all i∈N, if $g_{j,i}=\frac{\gamma_{t(j)+i-j-1}-\gamma_{t(j)+i-j}}{\gamma_{t(j)}}$, i>j, where $t\left(j\right)=1+\sum_{k<j}\left(1-U_{k}\right)$.

Lemma 5.1 shows that ${\text{ADDIS-Spending}}_{\text{local}}$ can be represented as an ADDIS‐Graph, in which significance level is distributed to all future hypotheses, even to the dependent ones, but only levels that come from independent hypotheses are used to test a hypothesis. It is intuitive, that it is more efficient to directly distribute significance level only to independent hypotheses.

For example, consider the parameters given in Example 3.3 with $L_{2}=1$ and $L_{3}=0$. It is easy to check that in this case $\alpha_{1}^{\text{spend}}=\alpha /4$, $\alpha_{2}^{\text{spend}}=\alpha /8$, and $\alpha_{3}^{\text{spend}}=\alpha /8$. However, we have shown in Figure 5 that the (specific) ADDIS‐Graph would yield in the same example $\alpha_{1}=\alpha /4$, $\alpha_{2}=\alpha /8$, and $\alpha_{3}=3\alpha /16$ (and one can check that also all future levels are greater with the ADDIS‐Graph), since it does not lose significance level due to conflicts. In the following proposition, we introduce a general algorithm exploiting this to uniformly improve the ${\text{ADDIS-Spending}}_{\text{local}}$.

Proposition 5.2 Let ${\left(\gamma_{i}\right)}_{i\in N}$ be nonincreasing and define $X_{i}=\left\{i-1,\text{…},i-L_{i}\right\}$. Then there exists a choice of weights ${\left(g_{j,i}^{*}\right)}_{j\in N,i>j}$ (see Algorithm S.1) such that the ${\text{ADDIS-Graph}}_{\text{conf}}$ uniformly improves the ${\text{ADDIS-Spending}}_{\text{local}}$. We denote this procedure as ${\text{ADDIS-Graph}}_{\text{conf-u}}$.

## 6 Extensions of the ADDIS‐Graph

### 6.1 Control of the Per‐Family Error Rate (PFER)

Tian and Ramdas (2021) showed that procedures satisfying (1) even control the more conservative PFER defined by

(7) $$\begin{array}{c} \text{PFER}≔E[v], \end{array}$$

where v is the number of false rejections. Hence, it is not an actual extension of the ADDIS‐Graph, but an immediate consequence of the result by Tian and Ramdas (2021), that the ADDIS‐Graph also controls the PFER at significance level α. It follows by 1{v>0}≤v that control of the PFER implies strong control of the FWER (see (10) for another explanation).

While we focused on the FWER since it is the more common error rate in practice, the PFER still offers good interpretability and for this reason there are also applications where the PFER is desirable. For example, in a platform trial many treatment groups are compared to the same control group. Now, if the control group performed badly, meaning worse outcomes were observed than usual, there is a danger of deeming many treatments as efficient even though they are not. The FWER does not protect against this case, as it only ensures that the probability of committing any type I error is small. However, if we are in the case of a type I error, it has no guarantee about the number of type I errors. This can be resolved by controlling the PFER and is therefore automatically provided by the ADDIS‐Graph. This example is not intended to question the appropriateness of the FWER for platform trials, but only to show that the control of the PFER provides additional sensible control.

### 6.2 Comparison to Closed ADDIS‐Spending and the Closed ADDIS‐Graph

Fischer et al. (2024a) introduced another improvement of the ADDIS‐Spending under local dependence based on their online closure principle. The *closed ADDIS‐Spending* tests each individual hypothesis at the level

(8) $$\begin{array}{cc} & \alpha_{i}^{\text{c-spend}}=\alpha \left(\tau_{i}-\lambda_{i}\right)\gamma_{t{\left(i\right)}^{\text{c-loc}}},\\ & \text{where}\;t{\left(i\right)}^{\text{c-loc}}=1+\sum_{j=i-L_{i}}^{i-1}\left(1-R_{j}\right)+\sum_{j=1}^{i-L_{i}-1}\left(1-\max\left\{U_{j},R_{j}\right\}\right), \end{array}$$

where $R_{j}=1\left\{P_{j}\leq \alpha_{j}^{\text{c-spend}}\right\}$. Note that this is a uniform improvement of the ADDIS‐Spending under local dependence (6), because $\sum_{j=i-L_{i}}^{i-1}\left(1-R_{j}\right)\leq L_{i}$ and $\max\left\{U_{j},R_{j}\right\}\geq U_{j}$. We usually choose $\lambda_{i}\geq \alpha_{i}$ (Tian and Ramdas 2021) and also assume this in the following argumentation such that the latter inequality becomes an equation and the only improvement comes from the former inequality. Hence, the difference compared to the ADDIS‐Spending under local dependence is that even if $P_{j}$ and $P_{i}$, i>j, depend on each other, the level $\alpha_{i}$ is allowed to be adjusted to the information whether $P_{j}\leq \alpha_{j}$. Thus, if a hypothesis $H_{j}$ is rejected, the closed ADDIS‐Spending no longer loses significance level due to the local dependence, however, it still does if $\alpha_{j}<P_{j}\leq \lambda_{j}$ or $P_{j}>\tau_{j}$.

Hence, one difference to the uniform improvement obtained by the ${\text{ADDIS-Graph}}_{\text{conf}}$ introduced in Section 5 is that the closed ADDIS‐Spending only improves the ADDIS‐Spending in case of a rejection, while the ${\text{ADDIS-Graph}}_{\text{conf}}$ even improves the ADDIS‐Spending if $\alpha_{j}<P_{j}\leq \lambda_{j}$ or $P_{j}>\tau_{j}$ (and local dependence is present). This suggests that the uniform improvement obtained by the ${\text{ADDIS-Graph}}_{\text{conf}}$ is stronger, which is verified by simulations (see Supporting Information section S.4). Another difference between the ${\text{ADDIS-Graph}}_{\text{conf}}$ and closed ADDIS‐Spending is the construction. While the closed ADDIS‐Spending is based on applying the (online) closure principle (Fischer et al. 2024a; Marcus et al. 1976) directly to the ADDIS‐Spending, the ${\text{ADDIS-Graph}}_{\text{conf}}$ defines another way to exploit condition (1). The latter has the advantage that it additionally provides control of the PFER as discussed in Section 6.1, while the closure principle only guarantees FWER control. Furthermore, the closed ADDIS‐Spending cannot be formulated for general conflict sets and the ${\text{ADDIS-Graph}}_{\text{conf}}$ provides a higher flexibility in general.

Nevertheless, this poses the question of whether the ${\text{ADDIS-Graph}}_{\text{conf}}$ can also be improved by the (online) closure principle under local dependence. As for the ADDIS‐Spending, one can construct a closed ${\text{ADDIS-Graph}}_{\text{conf}}$ under local dependence that tests the individual hypothesis $H_{i}$ at the level

(9) $$\begin{array}{cc} \alpha_{i}^{\text{c-graph}}= & \left(\tau_{i}-\lambda_{i}\right)\left(\alpha \gamma_{i}+\sum_{j=i-L_{i}}^{i-1}g_{j,i}R_{j}\frac{\alpha_{j}}{\tau_{j}-\lambda_{j}}\right.\\ & \left.+\sum_{j=1}^{i-L_{i}-1}g_{j,i}\max\left\{R_{j},U_{j}\right\}\frac{\alpha_{j}}{\tau_{j}-\lambda_{j}}\right), \end{array}$$

where $R_{j}=1\left\{P_{j}\leq \alpha_{j}^{\text{c-graph}}\right\}$ and we usually assume that $\max\left\{R_{j},U_{j}\right\}=U_{j}$ as above. We provide the derivation of this closed ${\text{ADDIS-Graph}}_{\text{conf}}$ in the Supporting Information S.1. The closed ${\text{ADDIS-Graph}}_{\text{conf}}$ allows to distribute significance level to dependent hypotheses in the case of a rejection and to independent hypotheses if $P_{i}\leq \lambda_{i}$ or $P_{i}>\tau_{i}$. Since the ${\text{ADDIS-Graph}}_{\text{conf}}$ can only distribute significance level to independent hypotheses (in case of $P_{i}\leq \lambda_{i}$ or $P_{i}>\tau_{i}$), the closed ${\text{ADDIS-Graph}}_{\text{conf}}$ could be seen as a uniform improvement of it. However, $g_{j,i}$ is only allowed to depend on information that is independent of $P_{i}$. Hence, if $P_{j}$ and $P_{i}$ depend on each other, $g_{j,i}$ must be fixed before knowing the true value of $P_{j}$ and we must decide whether we want to distribute some of the significance level $\alpha_{j}^{\text{c-graph}}$ to dependent hypotheses (in case of a rejection) before knowing whether $H_{j}$ will be rejected. Since it is much more likely that $P_{j}\leq \lambda_{j}$ or $P_{j}>\tau_{j}$ than $P_{j}\leq \alpha_{i}^{\text{c-graph}}$, one usually chooses $g_{j,i}=0$ for all i>j with $i-L_{i}\leq j$ in order to obtain a high power. In this case, the closed ${\text{ADDIS-Graph}}_{\text{conf}}$ reduces to the usual ${\text{ADDIS-Graph}}_{\text{conf}}$ under conflict sets. The only situation in which we believe that the closed ${\text{ADDIS-Graph}}_{\text{conf}}$ would provide a real uniform improvement of the ${\text{ADDIS-Graph}}_{\text{conf}}$ is when we have the information that all future hypotheses are dependent on the current $P_{j}$. In this case, it would be best to choose $\lambda_{j}=0$ and $\tau_{j}=1$ such that the closed ${\text{ADDIS-Graph}}_{\text{conf}}$ reduces to the online version (Tian and Ramdas 2021; Fischer et al. 2024a) of the classical graphical approach by Bretz et al. (2009), while the ${\text{ADDIS-Graph}}_{\text{conf}}$ becomes the more conservative online Bonferroni adjustment (Foster and Stine 2008; Tian and Ramdas 2021).

### 6.3 Exploiting the Joint Distribution of the p‐Values

In Section 6.1, we have noted that the ADDIS‐Graph even controls the more conservative error rate PFER and in Section 6.2 we have shown that the (online) closure principle does not give a direct improvement of the ADDIS‐Graph. Hence, the question is how the gap between PFER and FWER control can be used to improve the ADDIS‐Graph further. For this, note that the connection between the PFER and the FWER can also be explained by the Bonferroni inequality

(10) $$\begin{array}{cc} \text{FWER} & =P\left(\underset{i\in I_{0}}{⋃}\left\{P_{i}\leq \alpha_{i}\right\}\right)\leq \sum_{i\in I_{0}}P\left(P_{i}\leq \alpha_{i}\right)\\ & =\sum_{i\in I_{0}}E\left[1\left\{P_{i}\leq \alpha_{i}\right\}\right]=E\left[v\right]=\text{PFER}. \end{array}$$

It is known that the Bonferroni inequality leads to conservative procedures as it makes worst case assumptions about the joint distribution of the p‐values. Hence, one approach to improve the ADDIS‐Graph would be to incorporate information about the joint distribution of the p‐values. Note that such information is not always available. However, for example in a platform trial, the entire dependency between the p‐values comes from the shared control data and therefore can be determined if the number of observations shared is available. In the Supporting Informtion S.2, we demonstrate how such information about the correlation structure can be included in the ${\text{ADDIS-Graph}}_{\text{conf}}$ and compare it via simulations to the usual ${\text{ADDIS-Graph}}_{\text{conf}}$ in the Supporting Information section S.4. However, note that the proposed method only works if $\tau_{i}=1$ and the local dependence structure is given by batches.

### 6.4 ADDIS‐Graphs for FDR Control

While being the norm in validation studies (Wason et al. 2014), FWER control can be too conservative for certain applications, particularly if the number of hypotheses is large. Less conservative error rates often considered in the (online) multiple testing literature are the FDR introduced by Benjamini and Hochberg (1995) and the modified FDR (mFDR), where

(11) $$\begin{array}{cc} \text{FDR(}i)≔\; & E\left(\frac{\left|V(i)\right|}{|R(i)|\vee 1}\right)\\ \text{mFDR(}i)≔\; & \frac{E(|V(i)|)}{E(|R(i)|\vee 1)}\;\left(i\in N\right). \end{array}$$

The goal is to control FDR(i) or mFDR(i) at each time i∈N. While the FDR is the most common error rate in large‐scale multiple testing, mFDR control is often considered in online multiple testing since it is easier to prove and usually requires fewer assumptions (Foster and Stine 2008; Javanmard and Montanari 2018; Zrnic et al. 2021). Note that there is a strong connection to the PFER, which is basically the numerator of the mFDR. Hence, it is not surprising that PFER procedures can be extended comparatively easy to mFDR or even FDR control.

**FIGURE 6:**
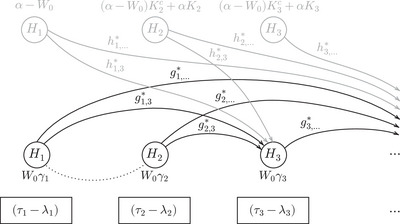
Illustration of the ${\text{FDR-ADDIS-Graph}}_{\text{conf}}$.

The ${\text{FDR-ADDIS-Graph}}_{\text{conf}}$, our ADDIS‐Graph for FDR control, is illustrated in Figure 6. The lower part (printed in black) can be interpreted exactly as the ADDIS‐Graph for FWER control (Figure 3). Furthermore, the weights ${\left(h_{j,i}^{*}\right)}_{j\in N,i>j}$ have the same properties as ${\left(g_{j,i}^{*}\right)}_{j\in N,i>j}$ and the indicator $K_{i}$ ($K_{i}^{c}=1-K_{i}$) is given by $K_{i}=1$, if the first rejection happened within the first i−1 steps and by $K_{i}=0$, otherwise. In contrast to the black arrows, the gray arrows are activated if the corresponding hypothesis is rejected. Hence, the ${\text{FDR-ADDIS-Graph}}_{\text{conf}}$ allows to distribute an additional level α to the future hypotheses after each rejection (except for the first rejection). Since no significance level is gained for the first rejection, FDR procedures often assume that a lower overall significance level of $W_{0}\leq \alpha$ is available at the beginning of the testing process such that $\left(\alpha -W_{0}\right)$ can be gained after the first rejection. In the Supporting Information S.3, we show that this ${\text{FDR-ADDIS-Graph}}_{\text{conf}}$ satisfies an ADDIS condition for FDR control by Tian and Ramdas (2019). This implies that the ${\text{FDR-ADDIS-Graph}}_{\text{conf}}$ provably controls the mFDR without any additional assumptions (Zrnic et al. 2021) and the FDR under independence of the p‐values (Tian and Ramdas 2019).

The benefit of the ${\text{FDR-ADDIS-Graph}}_{\text{conf}}$ compared to the current state‐of‐art ADDIS procedure for FDR control, the ${\text{ADDIS}}^{*}$ algorithm (Tian and Ramdas 2019), is similar as for the ${\text{FDR-ADDIS-Graph}}_{\text{conf}}$ for FWER control and the ${\text{ADDIS-Spending}}_{\text{local}}$. Due to its graphical structure, the ${\text{FDR-ADDIS-Graph}}_{\text{conf}}$ is more flexible and easier to interpret. In particular, the dependencies between the previous test outcomes and individual significance levels become clearer. This might be even more important in the FDR case, as the ${\text{ADDIS}}^{*}$ is far more complex than the ${\text{ADDIS-Spending}}_{\text{local}}$. Although we do not show a uniform improvement theoretically, the intuition about the superiority of the ${\text{FDR-ADDIS-Graph}}_{\text{conf}}$ over the ${\text{ADDIS}}^{*}$ algorithm when conflicts are present is the same as in the FWER case: The ${\text{FDR-ADDIS-Graph}}_{\text{conf}}$ distributes the same amount of significance level under conflict sets, while the ${\text{ADDIS}}^{*}$ algorithm loses level systematically due to conflicts. In the Supporting Information section S.4, we verify this by means of simulations and quantify the resulting power difference. Furthermore, these graphical representations clarify the gain of switching from FWER to FDR control.

## 7 Simulations

In this section, we compare the power and FWER control of the ${\text{ADDIS-Graph}}_{\text{conf-u}}$ and ${\text{ADDIS-Spending}}_{\text{local}}$ under local dependence using simulated data. Simulations for the extended ADDIS‐Graphs introduced in Section 6 can be found in the Supporting Information section S.4.

We consider n=100 hypotheses to be tested, whose corresponding p‐values ${\left(P_{i}\right)}_{i\in \{1,\text{…},n\}}$ follow a batch dependence structure $B_{1},\text{…},B_{n/b}$ with the same batch‐size b∈{1,5,10,20} for every batch. That means $B_{j}=\left\{P_{(j-1)b+1},\text{…},P_{jb}\right\}$, j∈{1,…,n/b}, and all p‐values within a batch $B_{j}$ depend on each other, while p‐values from different batches are independent. Let $X_{(j-1)b+1:bj}={\left(X_{(j-1)b+1},\text{…},X_{bj}\right)}^{T}$ be b‐dimensional i.i.d random vectors that follow a multivariate normal distribution with mean μ and covariance matrix σ, where $\mu ={\left(0,\text{…},0\right)}^{T}\in R^{b}$ and $\Sigma ={\left(\sigma_{ik}\right)}_{i,k=1,\text{…},b}\in R^{b\times b}$ with $\sigma_{ii}=1$ and $\sigma_{ik}=\rho \in \left(0,1\right)$ for all i∈{1,…,b} and k≠i. For each $H_{i}$, i∈{1,…,n}, we test the null hypothesis $H_{i}:\mu_{i}\leq 0$ with $\mu_{i}=E\left[Z_{i}\right]$, where $Z_{i}=X_{i}+3$ with probability $\pi_{A}\in \left(0,1\right)$ and $Z_{i}=X_{i}+\mu_{N}$, $\mu_{N}<0$, otherwise. Since the test statistics follow a standard Gaussian distribution under the null hypothesis, a z‐test can be used. The parameter $\pi_{A}$ can be interpreted as the probability of a hypothesis being false and $\mu_{N}$ as the conservativeness of null p‐values (Tian and Ramdas 2021).

**FIGURE 7:**
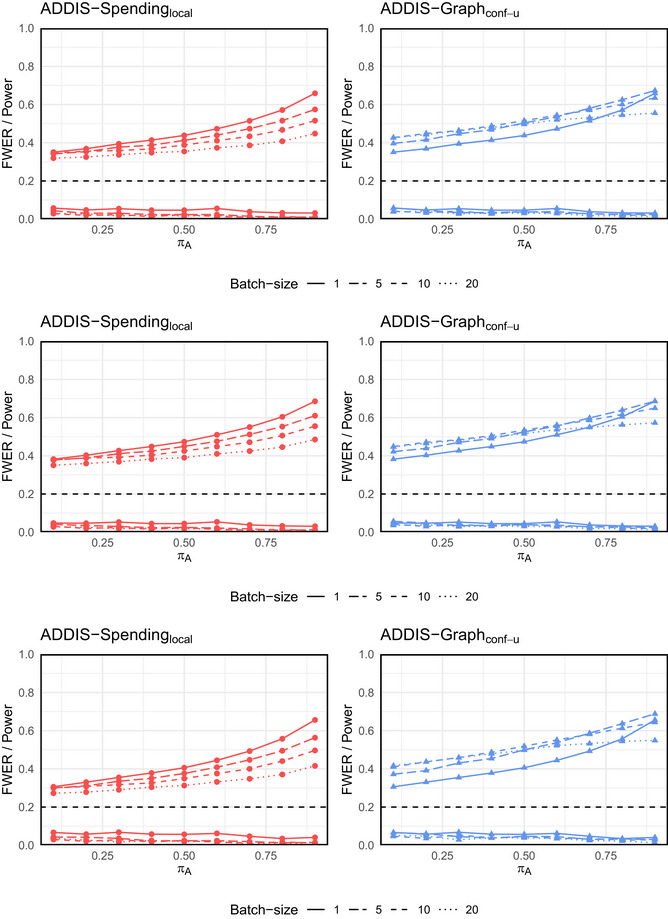
Comparison of ${\text{ADDIS-Spending}}_{\text{local}}$ and ${\text{ADDIS-Graph}}_{\text{conf-u}}$ in terms of power and FWER for different batch sizes and proportions of false null hypotheses ($\pi_{A}$). Lines above the overall level α=0.2 correspond to power and lines below to FWER. The p‐values were generated as described in the text with parameters $\mu_{N}=-0.5$ and ρ=0.5. Both procedures were applied with parameters $\tau_{i}=0.8$ and $\lambda_{i}=0.16$. In the top row $\gamma_{i}\propto 1/\left(\left(i+1\right)\log{\left(i+1\right)}^{2}\right)$, in the middle row $\gamma_{i}\propto 1/i^{1.6}$, and in the bottom row $\gamma_{i}=6/\left(\pi^{2}i^{2}\right)$. Under independence of the p‐values, both procedures coincide. However, the ${\text{ADDIS-Spending}}_{\text{local}}$ loses power when the p‐values become locally dependent, while the ${\text{ADDIS-Graph}}_{\text{conf-u}}$ offers a similar or even larger power as under independence.

We use an overall level α=0.2 and estimate the FWER and power of the ${\text{ADDIS-Graph}}_{\text{conf-u}}$ and ${\text{ADDIS-Spending}}_{\text{local}}$ (Tian and Ramdas 2021) by averaging over 1000 independent trials. Thereby, the proportion of rejected hypotheses among the false hypotheses is used as empirical power. We set $\mu_{N}=-0.5$ and ρ=0.5 in the simulations within this section, thus obtaining slightly conservative null p‐values with positive correlation within each batch. We provide results for other parameter configurations and time‐varying batch‐sizes in Supporting Information section S.4, which show a similar behavior of the two procedures across all considered settings. As recommended (Tian and Ramdas 2021), we choose $\tau_{i}=0.8$ and $\lambda_{i}=0.16$ for all i∈N. The rows in Figure 7 vary with respect to the chosen ${\left(\gamma_{i}\right)}_{i\in N}$, as the procedures are sensitive to it. In the top row, we use $\gamma_{i}\propto 1/\left(\left(i+1\right)\log{\left(i+1\right)}^{2}\right)$, in the middle row $\gamma_{i}\propto 1/i^{1.6}$, and in the bottom row $\gamma_{i}=6/\left(\pi^{2}i^{2}\right)$.

The results are provided in Figure 7. As shown in Lemma 5.1, the procedures are equivalent under independence of the p‐values. However, when the p‐values become locally dependent, the power of the ${\text{ADDIS-Spending}}_{\text{local}}$ decreases systematically in all cases, while the power of the ${\text{ADDIS-Graph}}_{\text{conf-u}}$ even increases in most cases. To understand why the power might increase under local dependence, note that the larger the batch size, the further into the future the significance level is distributed by the weights ${\left(g_{j,i}^{*}\right)}_{i=j+1}^{\infty }$ (see Algorithm S.1). This can lead to a more evenly distribution of the significance level under a larger batch size, which results in a higher power. However, if ${\left(\gamma_{i}\right)}_{i\in N}$ decreases slowly and the batch size is large, a lot of the significance level is distributed to hypotheses in the far future. Since the testing process is finite in this case, these hypotheses may never be tested, which is why power is lost when $\pi_{A}$ is large.

## 8 Application to RECOVERY Trial

**FIGURE 8:**
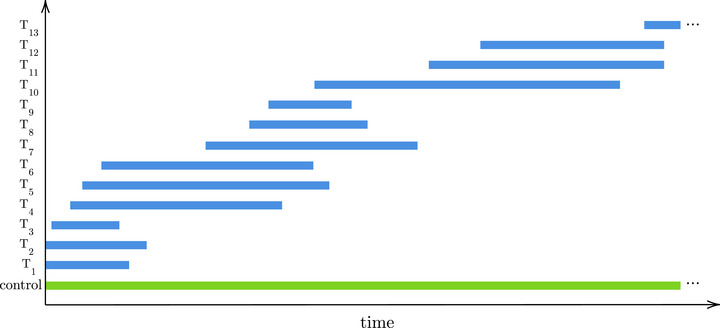
Overlapping structure of the RECOVERY trial (Sandercock et al. 2022).

In this section, we illustrate the usage of the ADDIS‐Graph by applying it on a real ongoing platform trial. The Randomised Evaluation of COVID‐19 Therapy (RECOVERY) trial was launched in 2020 and evaluates treatments for severe COVID‐19 diseases against a standard of care. Up to this date, 12 treatments have already been tested, while a 13th treatment is currently recruiting (Sandercock et al. 2022). The p‐values are reported at the website https://www.recoverytrial.net/. The platform trial structure is illustrated in Figure 8 and was copied from a publication by the data monitoring committee (Sandercock et al. 2022). As exemplified in Figure 2, overlapping hypotheses share some control data and are therefore conflicting. The ADDIS‐Graph can be used to adapt to these conflict sets. For example, the treatment arms $T_{1}$, $T_{2}$, and $T_{3}$ only distribute significance level to the treatment $T_{7}$ and onward, $T_{4}$ and $T_{6}$ distribute level to the treatment $T_{10}$ and onward, and $T_{5}$ to treatment $T_{11}$ and onward.

### Choice of the hyperparameters

When constructing an ADDIS‐Graph, there are many parameter choices one has to make; $\lambda_{i}$, $\tau_{i}$, $\gamma_{i}$, $g_{j,i}^{*}$. Based on this real platform trial, we will briefly describe the criteria that can be used to select them.

As observed by Tian and Ramdas (2019), it is theoretically desirable to choose $\lambda_{i}$ and $\tau_{i}$ such that the expectation of the left side in (1) is minimized, which is equivalent to minimizing $P\left(\lambda_{i}<P_{i}\leq \tau_{i}\right)/\left(\tau_{i}-\lambda_{i}\right)$ at each time i∈N. Since the true distribution of $P_{i}$ is usually unknown, in practice one often sets $\lambda_{i}=\lambda$ and $\tau_{i}=\tau$ for some constants 0≤λ<τ≤1. Tian and Ramdas (2021, 2019) proposed τ=0.8 and λ=0.16 in the FWER case and τ=0.5 and λ=0.25 in the FDR case. We have generally found ADDIS procedures to be quite robust against the choice of τ and λ as long as λ is not too small (<0.1) and τ not too large (>0.9) such that the ADDIS effect—discarding conservative null p‐values and adapting to the proportion of nonnull p‐values—can be realized. Therefore, we choose τ=0.8 and λ=0.3 to obtain the simple testing factor of τ−λ=0.5 in this application.

ADDIS procedures are more sensitive to the weights ${\left(\gamma_{i}\right)}_{i\in N}$ and ${\left(g_{j,i}^{*}\right)}_{j\in N,i>j}$. Let us first consider ${\left(\gamma_{i}\right)}_{i\in N}$. The parameter $\gamma_{i}$ sets the initial significance level for $H_{i}$, meaning $H_{i}$ will always be tested at least at the level $\alpha \left(\tau -\lambda \right)\gamma_{i}$. An important factor to consider when setting ${\left(\gamma_{i}\right)}_{i\in N}$ is approximately how many hypotheses are to be tested. For example, $\gamma_{1}=1/2$ might be reasonable if we only expect to test a few hypotheses, but risking to lose (if $\lambda <P_{1}\leq \tau$) one‐half of the significance level on the first hypothesis would be irresponsible when dozens of hypotheses are tested. Furthermore, one could choose ${\left(\gamma_{i}\right)}_{i\in N}$ based on contextual information to achieve a certain weighting between different hypotheses. For example, the inclusion of the first three treatment arms of the RECOVERY trial might have been planned at the same time. In this case, one could think of weighting these three treatments equally by choosing $\gamma_{1}=\gamma_{2}=\gamma_{3}$.

Designing a clinical trial is inherently challenging, as it requires making numerous assumptions—especially in the case of platform trials, which add further complexity. In retrospect, it is difficult to determine what information was available at the outset of the platform trial. Moreover, we lack detailed knowledge of the specific background behind each individual treatment that will enter the platform. We therefore choose a simple geometric sequence $\gamma_{i}=q^{i}\frac{1-q}{q}$, i∈N, for our application. The reason we go for a geometric sequence is that it is easy to interpret. The larger the parameter q, the slower ${\left(\gamma_{i}\right)}_{i\in N}$ converges to 0, and thus the better it can cover a large number of hypotheses. In addition, one can easily calculate the proportion of level spent after i hypotheses in the worst case (that all p‐values lie in (λ,τ])) by $\sum_{j=1}^{i}\gamma_{i}=1-q^{i}$. In order to take into account different possible prior information of the trialists, we apply the methods for different parameters q∈{0.6,0.7,0.8}.

As with ${\left(\gamma_{i}\right)}_{i\in N}$, the weights ${\left(g_{j,i}^{*}\right)}_{j\in N,i>j}$ can be chosen according to contextual information, such as study objectives, in practice, just like the classical graphical approach (Bretz et al. 2009). For example, there might be a relation between certain treatments $T_{j}$ and $T_{i}$, j<i, such that the level of $H_{j}$ should only be distributed to $H_{i}$ and not to any other hypothesis, which could be easily incorporated in the ADDIS‐Graph. However, if such information is not available, it is reasonable to just use ${\text{ADDIS-Graph}}_{\text{conf-u}}$, which chooses ${\left(g_{j,i}^{*}\right)}_{j\in N,i>j}$ based on ${\left(\gamma_{i}\right)}_{i\in N}$ and the conflict sets ${\left(X_{i}\right)}_{i\in N}$ in a way that leads to a uniform improvement of the ${\text{ADDIS-Spending}}_{\text{local}}$ (see Proposition 5.2 and Algorithm S.1 for the specific weights). This is also what we do in this application.

### Results

In Table 1, we compare the obtained rejections and the remaining significance level for future testing when applying the ${\text{ADDIS-Spending}}_{\text{local}}$ and the ${\text{ADDIS-Graph}}_{\text{conf-u}}$. In addition, we provide the results for an uncorrected procedure which tests each hypothesis at full level α=0.05 as a reference. The level for future hypotheses was calculated as the sum of future significance levels if a Bonferroni adjustment would be applied, meaning if one sets $\tau_{i}=1$ and $\lambda_{i}=0$, i>12. The results show that the ${\text{ADDIS-Graph}}_{\text{conf-u}}$ was able to reject one more hypothesis compared to ${\text{ADDIS-Spending}}_{\text{local}}$ in case of q=0.6. In addition, ${\text{ADDIS-Graph}}_{\text{conf-u}}$ leaves considerably more level for future hypotheses such that it is much more likely to obtain additional rejections in the future than with the ${\text{ADDIS-Spending}}_{\text{local}}$. Furthermore, the ${\text{ADDIS-Graph}}_{\text{conf-u}}$ appears to be more robust against the choice of q.

**TABLE 1:** Number of rejections and level for future hypotheses obtained by different procedures applied on the RECOVERY trial.

Procedure	Number of rejections			Level for future hypotheses		
	q=0.6	q=0.7	q=0.8	q=0.6	q=0.7	q=0.8
${\text{ADDIS-Spending}}_{\text{local}}$	2	3	3	0.0039	0.0084	0.0164
${\text{ADDIS-Graph}}_{\text{conf-u}}$	3	3	3	0.0256	0.0246	0.0263
Uncorrected	5	5	5	∞	∞	∞

## 9 Discussion

In their review paper, Robertson et al. (2023b) named the construction of online procedures for a small number of hypotheses and with known correlation structure, especially with respect to platform trials, as one of the future directions in online multiple testing. In addition, they claimed that the individual significance levels assigned by asynchronous online procedures are more conservative. In this paper, we constructed ADDIS‐Graphs that, due to their graphical structure, perfectly adapt to such complex trial designs (see, e.g., Figure 2). We demonstrated that the ADDIS‐Graphs lead to power improvements over the current state‐of‐art methods, as the level that is lost due to pessimistic assumptions because of local dependence or asynchronous testing, is reused at later steps, such that no significance level is lost overall. In particular, we showed that the ADDIS‐Graph for FWER control uniformly improves the ADDIS‐Spending under local dependence (Tian and Ramdas 2021). Due to their graphical structure (Bretz et al. 2009), ADDIS‐Graphs are flexible and easily comprehensible—and therefore facilitate the planning and conduction of a trial. For example, when the same sponsors run several treatment arms in a platform trial, they may want that significance level is only distributed between their hypotheses, which could easily be incorporated by an ADDIS‐Graph, but not by the ADDIS‐Spending.

We introduced several extensions of the ADDIS‐Graph for FWER control. First, we showed how the online closure principle (Fischer et al. 2024a) can be used to improve the ADDIS‐Graph in situations where all future p‐values depend on the current one. Moreover, we demonstrated how information about the joint distribution of the p‐values can be incorporated to improve the procedure, which is particularly relevant for platform trials. Furthermore, we presented an ADDIS‐Graph for FDR control.

Our proposed method for incorporating the joint distribution while adapting to the number of false hypotheses only allows to exploit the correlation structure within batches and therefore does not unlock the full potential of the approach. We wonder whether it is possible to exhaust the entire information about the joint distribution while still adapting to the number of false hypotheses. Also, it would be interesting to additionally discard the conservative null p‐values. An approach could be given in our previous work (Fischer et al. 2024b), where we introduced an improvement of the ADDIS condition (1) under independence of the p‐values by exploiting this specific dependence structure. However, we have not yet found a way to extend this idea to locally dependent p‐values.

Moreover, we argued that the FDR‐ADDIS‐Graph is superior to the ${\text{ADDIS}}^{*}$ algorithm for similar reasons as in the FWER case, which was also verified by simulations. However, we did not prove a uniform improvement for a specific choice of weights, which would be an interesting question for future work. Similarly, it remains open whether the FDR‐ADDIS‐Graph contains all procedures satisfying ADDIS condition for FDR control (Tian and Ramdas 2019), as we only proved this for the FWER case.

Another interesting task for future work would be to derive a theoretically grounded choice for the initial weights ${\left(\gamma_{i}\right)}_{i\in N}$, which occur not only in the ADDIS‐Graph but also in many other online procedures (Tian and Ramdas 2021, 2019; Ramdas et al. 2018; Javanmard and Montanari 2018).

## Conflicts of Interest

The authors declare no conflicts of interest.

## Open Research Badges

This article has earned an Open Data badge for making publicly available the digitally‐shareable data necessary to reproduce the reported results. The data is available in the Supporting Information section.

This article has earned an open data badge “**Reproducible Research**” for making publicly available the code necessary to reproduce the reported results. The results reported in this article could fully be reproduced.

## Supporting information


**Supporting File 1:** bimj70075‐sup‐0001‐SuppMat.pdf.

**Supporting File 2:** bimj70075‐sup‐0002‐DataCode.zip.

## Data Availability

The data that support the findings of this study are available in the Supporting Information of this article.
