# Supplementary material for: ADDIS‐Graphs for Online Error Control With Application to Platform Trials
Source: Biom J. 2025 Sep 28;67(5):e70075. doi: 10.1002/bimj.70075 (PMC12476829; doi:10.1002/bimj.70075)
Supplement: Supplementary file 1 — Supporting File 1: bimj70075‐sup‐0001‐SuppMat.pdf. [file BIMJ-67-e70075-s002.pdf]

Biometrical Journal **52** (2010) 61, zzz–zzz / DOI: 10.1002/bimj.200100000

Supplementary material for "ADDIS-Graphs for online error control with application to platform trials"

Lasse Fischer[*,1], **Marta Bofill Roig** [2], and **Werner Brannath** [1]

[1] Competence Center for Clinical Trials Bremen, University of Bremen, Bremen, Germany
[2] Department of Statistics and Operations Research, Universitat Politècnica de Catalunya – BarcelonaTech, Barcelona, Spain

S.1 Derivation of the closed ADDIS-Graph

In this section we derive the closed ADDIS-Graph$_{\text{conf}}$ (9) as an online closed procedure (Fischer et al. 2024). We use a similar construction of the closed procedure to that Fischer et al. (2024) did for the Online-Graph. For this, let a local dependence structure $\mathcal{X}_i = \{i-1, \dots, i-L_i\}$ be given, where $(L_i)_{i\in\mathbb{N}}$ are lags with $L_{i+1} \leq L_i + 1$. Furthermore, let $(\tau_i)_{i\in\mathbb{N}}$, $(\lambda_i)_{i\in\mathbb{N}}$, $(\gamma_i)_{i\in\mathbb{N}}$ and $(g_{j,i})_{j\in\mathbb{N},i>j}$ be sequences as in Definition 3.1 such that τ_i, λ_i, γ_i and $g_{j,i}$ are measurable with respect to $\mathcal{G}_{-\mathcal{X}_i}$. For each $I \subseteq \mathbb{N}$, we define an intersection test ϕ_I as

$$\phi_I = \mathbb{1}\left\{\exists i \in I : P_i \leq \alpha_i^I\right\},$$

$$\text{where } \alpha_i^I = (\tau_i - \lambda_i)\left(\alpha\gamma_i + \sum_{j\in I, j<i-L_i} g_{j,i}U_j \frac{\alpha_j^I}{\tau_j - \lambda_j} + \sum_{j\notin I, j<i} g_{j,i} \frac{\alpha_j^{I\cup\{j\}}}{\tau_j - \lambda_j}\right).$$

It holds that $\sum_{j\leq i, j\in I} \frac{\alpha_j^I}{\tau_j - \lambda_j}(1-U_j) \leq \alpha$ for all $i \in \mathbb{N}$, since for all indices that are not contained in I, we just shift the significance level to the future hypotheses according to the weights $(g_{j,i})_{j\in\mathbb{N},i>j}$. With this, it follows that ϕ_I, $I \subseteq \mathbb{N}$, is an α-level intersection test, meaning $\mathbb{P}_{H_I}(\phi_I = 1) \leq \alpha$, where $H_I = \bigcap_{i\in I} H_i$. Furthermore, the family of intersection tests $(\phi_I)_{I\subseteq\mathbb{N}}$ is consonant and predictable (Fischer et al. 2024). With this, Theorem 4.2 by Fischer et al. (2024) implies that the corresponding closed procedure is defined by the individual significance levels $(\alpha_i^{I_i})_{i\in\mathbb{N}}$, where $I_1 = \{1\}$ and $I_i = \{j \in \mathbb{N} : j < i, P_j > \alpha_j^{I_j}\} \cup \{i\}$ for all $i \geq 2$. This can also be written as

$$\begin{aligned}
&\alpha_i^{\text{c-graph}} \\
&= \alpha_i^{I_i} \\
&= (\tau_i - \lambda_i)\left(\alpha\gamma_i + \sum_{j\in I_i, j<i-L_i} g_{j,i}U_j \frac{\alpha_j^{I_i}}{\tau_j - \lambda_j} + \sum_{j\notin I_i, j<i} g_{j,i} \frac{\alpha_j^{I_i\cup\{j\}}}{\tau_j - \lambda_j}\right) \\
&= (\tau_i - \lambda_i)\left(\alpha\gamma_i + \sum_{j<i-L_i} g_{j,i}(1-R_j)U_j \frac{\alpha_j^{\text{c-graph}}}{\tau_j - \lambda_j} + \sum_{j<i} g_{j,i}R_j \frac{\alpha_j^{\text{c-graph}}}{\tau_j - \lambda_j}\right) \\
&= (\tau_i - \lambda_i)\left(\alpha\gamma_i + \sum_{j=1}^{i-L_i-1} g_{j,i} \max\{R_j, U_j\} \frac{\alpha_j^{\text{c-graph}}}{\tau_j - \lambda_j} + \sum_{j=i-L_i}^{i-1} g_{j,i}R_j \frac{\alpha_j^{\text{c-graph}}}{\tau_j - \lambda_j}\right),
\end{aligned}$$

*Lasse Fischer: e-mail: fischer1@uni-bremen.de, Phone: +49-421-218-63786

© 2010 WILEY-VCH Verlag GmbH & Co. KGaA, Weinheim

where $R_j = \mathbb{1}\{P_j \leq \alpha_j^{\text{c-graph}}\}$.

S.2 Exploiting the correlation structure when considering FWER control

When information about the joint distribution of p-values is available, ignoring this information might result in a conservative procedure (Westfall and Young 1993). Therefore, in this section we aim to incorporate such information into ADDIS procedures under local dependence. However, in order to do so we have to make some assumptions. First, we restrict to $\tau_i = 1$ for all $i \in \mathbb{N}$. Therefore, we only adapt to the proportion of false null hypotheses and skip the discarding of conservative null p-values. Furthermore, we assume that the hypotheses follow a batch dependence structure (see Section 5 for further explanation). We denote b_i, $i \in \mathbb{N}$, as the index of the batch that contains the p-value P_i. At last, we assume that the subset pivolatity condition holds (Westfall and Young 1993), which states that the distribution of $\mathbf{P_I}|H_I$ is the same as $\mathbf{P_I}|H_\mathbb{N}$ for every $I \subseteq \mathbb{N}$, where $\mathbf{P_I} = (P_i)_{i\in I}$ is a random vector of p-values. This is a very common assumption made when incorporating information about the joint distribution of p-values into multiple testing procedures and shown to hold in a wide range of settings, e.g. in a multivariate Gaussian (Westfall and Young 1993).

Theorem S.1 *Assume that the subset pivotality condition is satisfied. Let the local dependence structure be given by batches* $(B_i)_{i\in\mathbb{N}}$*. Furthermore, let* $\lambda_{b_i} \in [0,1)$ *and* α_i *be measurable regarding* $\mathcal{F}_{b_i-1} = \sigma(\{P_j\}_{j:b_j<b_i})$ *for all* $i \in \mathbb{N}$*, where* $R_j = \mathbb{1}\{P_j \leq \alpha_j\}$ *and* $C_j = \mathbb{1}\{P_j \leq \lambda_{b_j}\}$*. Every multiple testing procedure controls the FWER in the strong sense when the individual significance levels* $(\alpha_i)_{i\in\mathbb{N}}$ *satisfy*

$$\sum_{j=1}^{i} \frac{\alpha_j^c}{1-\lambda_{b_j}}(1-C_j) \leq \alpha \quad \textit{for all } i \in \mathbb{N}, \tag{S.1}$$

where $\alpha_j^c := \mathbb{P}_{H_\mathbb{N}}\left(\bigcap_{k\in B_{b_j},k<j,C_k=0}\{P_k > \alpha_k\} \cap \{P_j \leq \alpha_j\} \middle| \mathcal{F}_{b_j-1}\right)$ *and* $\mathbb{P}_{H_\mathbb{N}}$ *indicates that we calculate the probability under the global null hypothesis.*

Remark S.2 Note that this is a uniform improvement of the classical ADDIS principle under local dependence (Tian and Ramdas 2021) for $\tau_i = 1$ and batch-wise fixed $\lambda_i = \lambda_{b_i}$, since $\alpha_i^c \leq \alpha_i$ for all $i \in \mathbb{N}$.

We propose the following Adaptive-Graph as example procedure that satisfies Theorem S.1.

Definition S.3 (Adaptive-Graph$_{\text{corr}}$) Assume the local dependence structure is given by the batches $(B_i)_{i\in\mathbb{N}}$. Let $\lambda_{b_i} \in [0,1)$, $(\gamma_i)_{i\in\mathbb{N}}$ be a non-negative sequence that sums up to 1 and $(g_{j,i}^*)_{i=j+1}^{\infty}$ be a non-negative sequence for all $j \in \mathbb{N}$ such that $g_{j,i}^* = 0$ if $b_j = b_i$ and $\sum_{i:b_i>b_j}^{\infty} g_{j,i}^* \leq 1$. In addition, let λ_{b_i}, γ_i and $g_{j,i}^*$, be measurable regarding $\mathcal{F}_{b_i-1}$. The Adaptive-Graph$_{\text{corr}}$ tests each hypothesis H_i at significance level

$$\alpha_i = (1-\lambda_{b_i})\left(\alpha\gamma_i + \sum_{j:b_j<b_i} g_{j,i}^* C_j \frac{\alpha_j}{1-\lambda_{b_j}} + \sum_{j:b_j<b_i} g_{j,i}^*(1-C_j)\frac{\alpha_j-\alpha_j^c}{1-\lambda_{b_j}}\right). \tag{S.2}$$

Theorem S.4 *The Adaptive-Graph*$_{corr}$ *satisfies Theorem S.1 and thus controls the FWER strongly when the subset pivotality condition is satisfied.*

The Adaptive-Graph$_{\text{corr}}$ can be interpreted just as the ADDIS-Graph$_{\text{conf}}$ (Figure 5), however, if $P_i > \lambda_{b_i}$, the significance level $\alpha_i - \alpha_i^c$ is additionally distributed to the future hypotheses.

Remark S.5 In general, exploiting correlation structures in graphical test procedures is not straightforward, as the required consonance can get lost (Bretz et al. 2011). However, in the above described batch

© 2010 WILEY-VCH Verlag GmbH & Co. KGaA, Weinheim

dependence setting, the here introduced Adaptive-Graph$_{\text{corr}}$ brings together the graphical approach and the utilization of information about the joint distribution of p-values.

Remark S.6 If one chooses $B_1 = \{P_1, P_2, \ldots\}$ and $\lambda_{b_1} = 0$, the Adaptive-Graph$_{\text{corr}}$ no longer adapts to the number of false hypotheses, however, it allows to exploit the joint distribution among all hypotheses. This can be useful if there are no or only very few independent p-values.

The batch setting assumed in Theorem S.1 may seems very restrictive. However, it arises naturally in a lot of settings. For example, if the data for testing the hypotheses is replaced by new, independent data after a period of time. This is e.g. the case when a machine learning algortihm is updated over time and after testing several modifications, a new evaluation data set is used for future modifications (Feng et al. 2021, 2022). Sometimes also platform trials are performed in a batch setting when multiple treatment arms enter and leave the trial at the same time (Robertson et al. 2023). But also if this is not the case, platform trials can still be transformed into such a batch setting. For this, we specify a local dependence structure for batches and adjust the procedures in the same way as shown before for single hypotheses. For example, one could batch the p-values from Figure 2 as $B_1 = \{P_1\}$, $B_2 = \{P_2, P_3, P_4, P_5\}$ and $B_3 = \{P_6, \ldots\}$. With this, the correlation within the batch B_2 could be exploited, but due to the dependence of P_1 and P_2 all the significance levels used for testing hypotheses in B_2 would not be allowed to use information about P_1. However, the significance levels for B_3 could depend on P_1. This could save a lot of significance level, particularly, if the testing process continues after T_6.

S.3 Extension to FDR control

Tian and Ramdas (2019) introduced the following ADDIS condition for FDR control

$$\frac{\sum_{j=1}^{i} \frac{\alpha_j}{\tau_j - \lambda_j}(1 - U_j)}{|R(i)| \vee 1} \leq \alpha \quad \text{for all } i \in \mathbb{N}. \tag{S.3}$$

The only difference to the ADDIS condition for the FWER control (1) is the denominator $|R(i)| \vee 1$. Bringing it on the other side, it can be interpreted as if an additional level α is gained after each rejection except for the first one. This can be incorporated into the ADDIS-Graph by distributing an additional α to future hypotheses in case of rejection according to non-negative weights $(h_{j,i})_{i=j+1}^{\infty}$ such that $\sum_{i=j+1}^{\infty} h_{j,i} \leq 1$ for all $j \in \mathbb{N}$. For example, one could just choose $h_{j,i} = g_{j,i}$.

Since no significance level is gained for the first rejection, FDR procedures often start with a lower overall significance level $W_0 \leq \alpha$ such that $(\alpha - W_0)$ can be gained after the first rejection. To differentiate between the first and other rejections, we additionally define the indicator K_i with $K_i = 1$, if the first rejection happened within the first $i-1$ steps and $K_i = 0$, otherwise. We also set $K_i^c = 1 - K_i$. With this, the ADDIS-Graph for FDR control can be defined as follows.

Definition S.1 (FDR-ADDIS-Graph$_{\text{conf}}$) Let the conflict sets be given by $(\mathcal{X}_i)_{i \in \mathbb{N}}$. Furthermore, let $(\gamma_i)_{i \in \mathbb{N}}$, $(g^*_{j,i})_{j \in \mathbb{N}, i > j}$, $(\tau_i)_{i \in \mathbb{N}}$ and $(\lambda_i)_{i \in \mathbb{N}}$ be as in ADDIS-Graph$_{\text{conf}}$ (Definition 4.1). In addition, let $W_0 \leq \alpha$ and $(h^*_{j,i})_{i=j+1}^{\infty}$, $j \in \mathbb{N}$, be a non-negative sequence such that $h^*_{j,i} = 0$ if $j \in \mathcal{X}_i$ and $\sum_{i>j, j \notin \mathcal{X}_i}^{\infty} h^*_{j,i} \leq 1$. The FDR-ADDIS-Graph$_{\text{conf}}$ tests each hypothesis H_i at significance level $\alpha_i = \min(\hat{\alpha}_i, \lambda_i)$, where

$$\hat{\alpha}_i = (\tau_i - \lambda_i) \left(W_0 \gamma_i + \sum_{j=1}^{i-1} g^*_{j,i} U_j \frac{\hat{\alpha}_j}{\tau_i - \lambda_j} + \sum_{j=1}^{i-1} h^*_{j,i} R_j [\alpha K_j + (\alpha - W_0) K_j^c] \right)$$

with $R_j = \mathbb{1}\{P_j \leq \alpha_j\}$.

© 2010 WILEY-VCH Verlag GmbH & Co. KGaA, Weinheim

In order to control the FDR using ADDIS procedures, α_i, λ_i and $1-\tau_i$, $i \in \mathbb{N}$, are required to be monotonic functions of the past (Tian and Ramdas 2019). This means that they are coordinatewise non-decreasing functions in $R_{1:(i-1)} := (R_1, \dots, R_{i-1})$ and $U_{1:(i-1)} := (U_1, \dots, U_{i-1})$. An easy way to satisfy this using the FDR-ADDIS-Graph$_{\text{conf}}$ is to choose the parameters λ_i, τ_i, γ_i, $g^*_{j,i}$ and $h^*_{j,i}$ for all $i \in \mathbb{N}, j < i$, independently of the past. Then α_i is a monotonic function of the past by definition.

Theorem S.2 *The FDR-ADDIS-Graph$_{conf}$ satisfies equation* (S.3)*. Thus, it controls the mFDR(i) for all $i \in \mathbb{N}$ (Zrnic et al. 2021). Furthermore, it controls the FDR(i) for all $i \in \mathbb{N}$ when α_i, λ_i and $1-\tau_i$ are monotonic functions of the past and the null p-values are independent from each other and the non-nulls (Tian and Ramdas 2019).*

S.4 Further simulation results

S.4.1 Comparison of the ADDIS-Graph and closed ADDIS-Spending under local dependence

In this subsection, we compare the closed ADDIS-Spending$_{\text{local}}$ (8) with the ADDIS-Graph$_{\text{conf-u}}$ (4). We use exactly the same simulation setup as in Section 7 and apply the procedures with the same parameters.

The results are illustrated in Figure S.1 and look very similar to Figure 7. The only difference is that in Figure S.1 the closed ADDIS-Spending$_{\text{local}}$ loses slightly less power due to the local dependence than the ADDIS-Spending$_{\text{local}}$. However, the ADDIS-Graph$_{\text{conf-u}}$ also outperforms the closed ADDIS-Spending$_{\text{local}}$ in all cases.

S.4.2 Comparison of the ADDIS-Graph and ADDIS-Spending under local dependence for other design parameters

In this section, we consider the same simulation setup as in Section 7 for $\gamma_i = 6/(\pi^2 i^2)$, $i \in \mathbb{N}$, but change the conservativeness of null p-values, strength of the within-batch correlation or the local dependence structure.

Conservativeness of null p-values. In Figure S.2, we compare the ADDIS-Spending$_{\text{local}}$ and the ADDIS-Graph$_{\text{conf-u}}$ for different $\mu_N \in \{0, -1, -2\}$. The results show a similar behavior to Figure 7; the ADDIS-Spending$_{\text{local}}$ loses power systematically for increasing batch-size, while the power of the ADDIS-Graph$_{\text{conf-u}}$ remains similar.

Within-batch correlation. In Figure S.2, we vary the within-batch correlation $\rho \in \{0.2, 0.8\}$. Again, the behavior is similar to Figure 7.

Time-varying batch-sizes. In Figure S.4, we consider batch-sizes that vary over time. A smallest batch-size of s means that we have batch sizes of $b \in \{s, 2s, 3s, 4s\}$ and all batch-sizes occur equally often. For example, in case of $s = 5$, we have two batches of size $b = 5$, two of size $b = 10$, two of size $b = 15$ and two of size $b = 20$. In the top row, the batch-size is increasing and and in the bottom row, the batch-size is decreasing. As before, the behavior is similar to Figure 7.

S.4.3 Simulation results when incorporating correlation structure

In this subsection, we use the same simulation setup as described in Section 7 to compare the ADDIS-Graph$_{\text{conf}}$ (4) with the Adaptive-Graph$_{\text{corr}}$ (S.2). The results are summarized in Figure S.5. In the top row, we vary the batch-size $b \in \{1, 5, 10, 20\}$, in the middle row the correlation within batches $\rho \in \{0.3, 0.5, 0.7, 0.9\}$ and in the bottom row, we evaluate the procedures for a different conservativeness of null p-values $\mu_N \in \{0, -0.5, -1, -2\}$, while the other parameters are set to standard values $b = 10$, $\rho = 0.5$ and $\mu_N = 0$. In

© 2010 WILEY-VCH Verlag GmbH & Co. KGaA, Weinheim

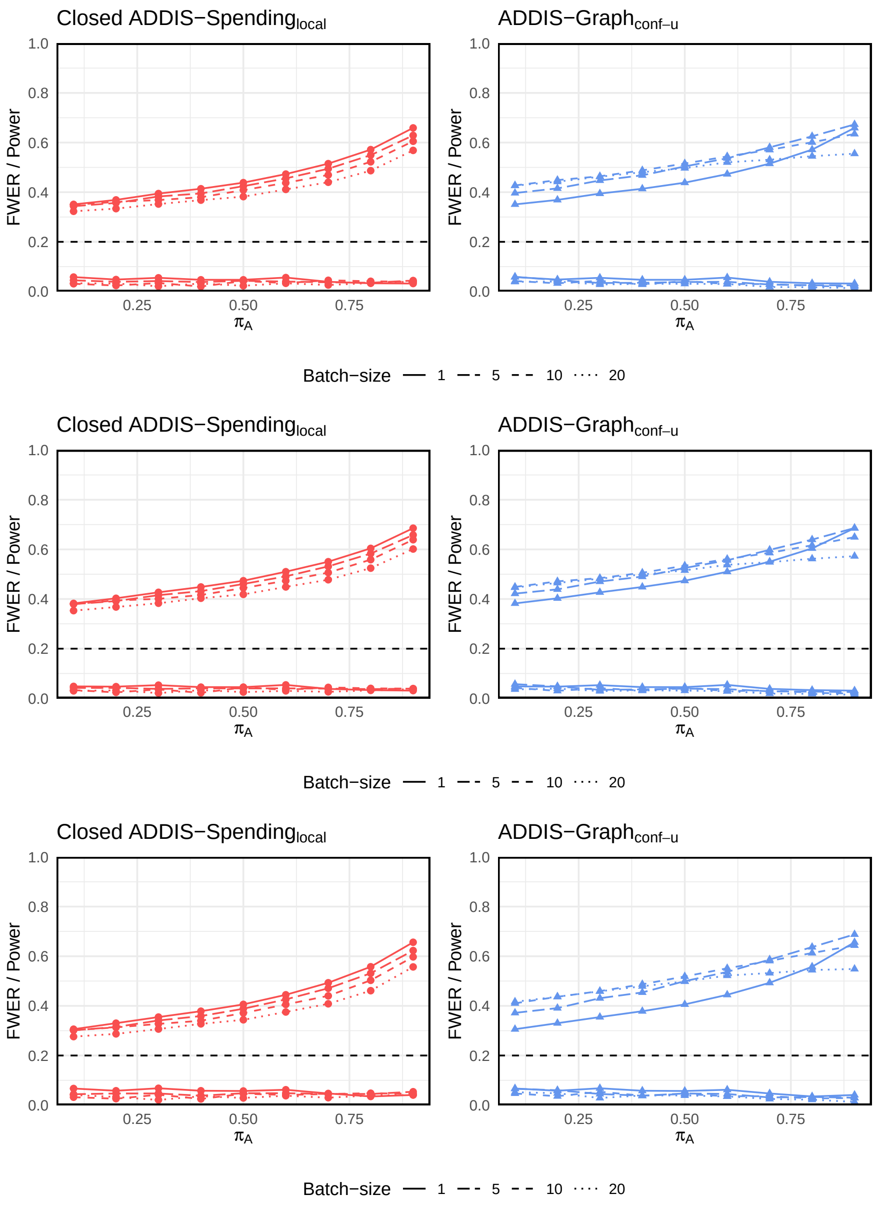

Figure S.1 Comparison of closed ADDIS-Spending$_{\text{local}}$ (8) and ADDIS-Graph$_{\text{conf-u}}$ (4) in terms of power and FWER for different batch-sizes and proportions of false null hypotheses (π_A). Lines above the overall level $\alpha = 0.2$ correspond to power and lines below to FWER. Both procedures were applied with parameters $\tau_i = 0.8$ and $\lambda_i = 0.16$. In the top row $\gamma_i \propto 1/\left((i+1)\log(i+1)^2\right)$, in the middle row $\gamma_i \propto 1/i^{1.6}$ and in the bottom row $\gamma_i = 6/(\pi^2 i^2)$. Under independence of the p-values both procedures coincide. However, the closed ADDIS-Spending$_{\text{local}}$ loses power when the p-values become locally dependent, while the ADDIS-Graph$_{\text{conf-u}}$ offers a similar or even larger power compared to under independence.

© 2010 WILEY-VCH Verlag GmbH & Co. KGaA, Weinheim

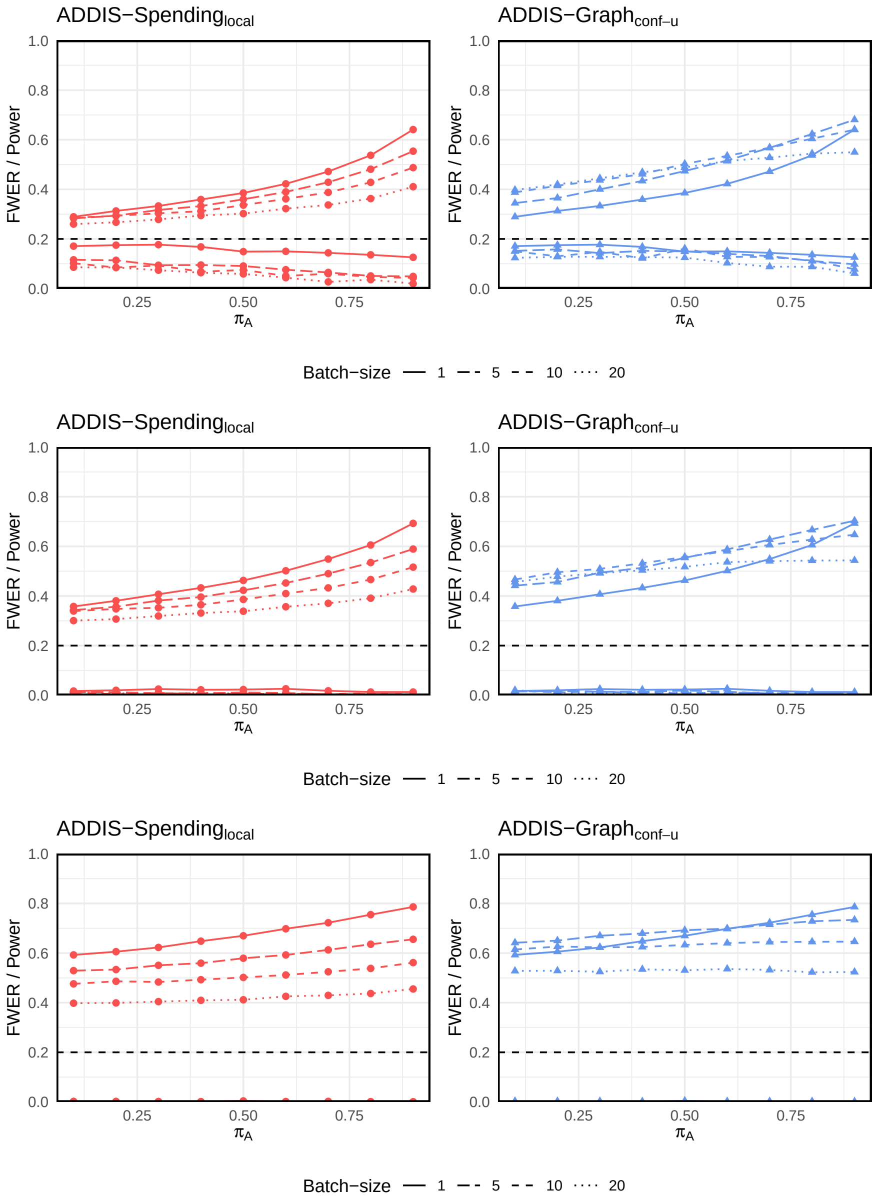

Figure S.2 Comparison of ADDIS-Spending$_{\text{local}}$ (6) and ADDIS-Graph$_{\text{conf-u}}$ (4) in terms of power and FWER for different batch-sizes and proportions of false null hypotheses (π_A). In the top row $\mu_N = 0$, in the middle row $\mu_N = -1$ and in the bottom row $\mu_N = -2$. As in Figure 7, ADDIS-Spending$_{\text{local}}$ loses power when the p-values become locally dependent, while the power of the ADDIS-Graph$_{\text{conf-u}}$ remains similar.

© 2010 WILEY-VCH Verlag GmbH & Co. KGaA, Weinheim

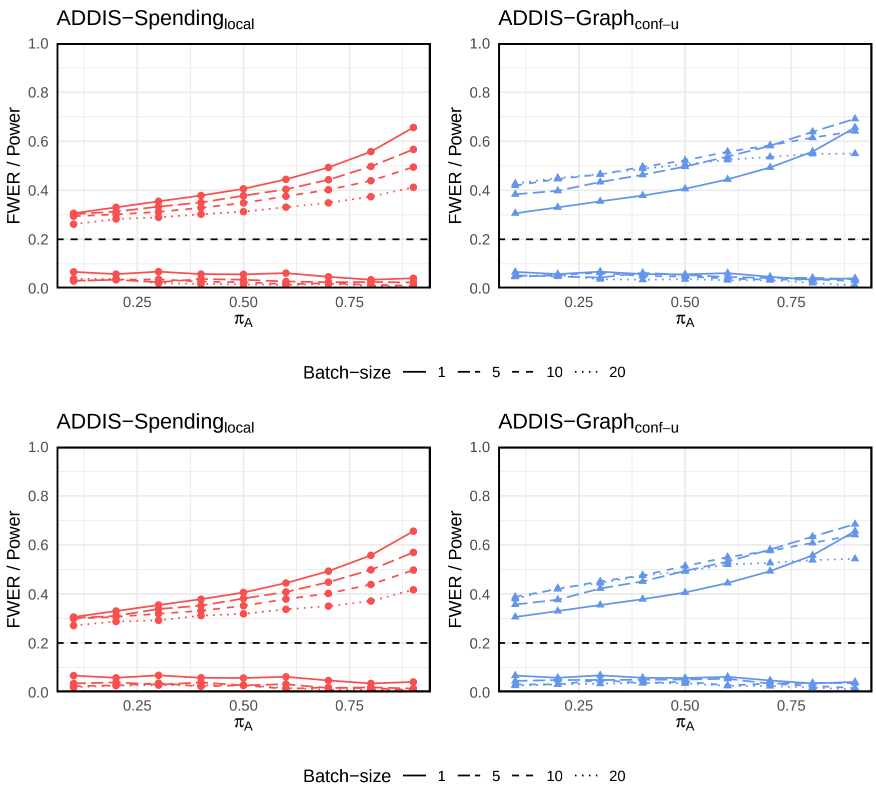

Figure S.3 Comparison of ADDIS-Spending$_{\text{local}}$ (6) and ADDIS-Graph$_{\text{conf-u}}$ (4) in terms of power and FWER for different batch-sizes and proportions of false null hypotheses (π_A). In the top row $\rho = 0.2$ and in the bottom row $\rho = 0.8$. As in Figure 7, ADDIS-Spending$_{\text{local}}$ loses power when the p-values become locally dependent, while the power of the ADDIS-Graph$_{\text{conf-u}}$ remains similar.

order to extract the effect of incorporating the correlation structure, we set $\tau_i = 1$ for the ADDIS-Graph$_{\text{conf}}$ in the top and middle row, which is why we also write Adaptive-Graph$_{\text{conf}}$. Furthermore, we choose $\lambda_i = \tau_i\alpha$, $\gamma_i = 6/(\pi^2 i^2)$ and $g^*_{j,i} = g_{j,i} \Big/ \left(1 - \sum_{k=j+1}^{d_j-1} g_{j,k}\right)$ if $i \geq d_j$ and $g^*_{j,i} = 0$ otherwise, where $g_{j,i} = \gamma_{i-j}$ and $d_j = \min\{i \in \mathbb{N} : i - L_i > j\}$, for both procedures in all cases.

In the top row, the two procedures are equivalent under independence. However, when the batch-size increases, the power of both procedures increases as well, while the power gain is larger using the Adaptive-Graph$_{\text{corr}}$. The plots looks different in the middle row, where the power of the Adaptive-Graph$_{\text{conf}}$ remains identical when varying the ρ, while the FWER drops a bit for large ρ. This FWER drop can be compensated by exploiting the correlation structure using Adaptive-Graph$_{\text{corr}}$. It also seems that the strength of correlation in the middle row has a larger positive impact on the power of the Adaptive-Graph$_{\text{corr}}$ than the batch-size in the top row. The comparison looks quite different in the bottom row, where conservative p-values are now discarded using the ADDIS-Graph$_{\text{conf}}$. When the null p-values are uniformly distributed ($\mu_N = 0$), the Adaptive-Graph$_{\text{corr}}$ is still superior, however, when the null p-values become conservative, the power of the ADDIS-Graph$_{\text{conf}}$ increases, while the Adaptive-Graph$_{\text{corr}}$ loses a bit of power. To conclude, it is difficult to give a general advice on whether one should prefer the Adaptive-Graph$_{\text{corr}}$ or

© 2010 WILEY-VCH Verlag GmbH & Co. KGaA, Weinheim

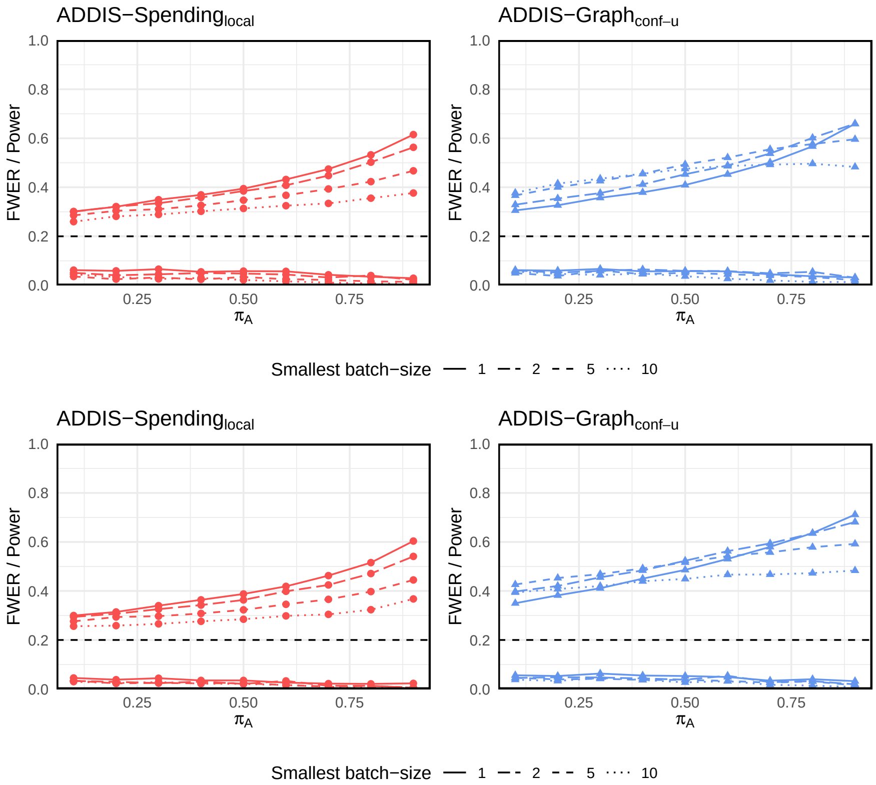

Figure S.4 Comparison of ADDIS-Spending$_{\text{local}}$ (6) and ADDIS-Graph$_{\text{conf-u}}$ (4) in terms of power and FWER for time-varying batch-sizes and different proportions of false null hypotheses (π_A). In the top row the batch-size is increasing and in the bottom row the batch-size is decreasing. As in Figure 7, ADDIS-Spending$_{\text{local}}$ loses power when the p-values become locally dependent, while the power of the ADDIS-Graph$_{\text{conf-u}}$ remains similar.

ADDIS-Graph$_{\text{conf}}$ and the choice of the procedure should incorporate information/assumptions about the batch-size, strength of correlation and conservativeness of null p-values.

S.4.4 Comparison of FDR-ADDIS-Graph and ADDIS* in an asynchrone test setup

In this subsection we consider a similar simulation setup as described in Section 7, but for independent p-values ($b = 1$). Applying the procedures, it is assumed that the hypotheses are tested in an asynchronous manner. Thus, the conflict sets are given by $\mathcal{X}_i = \{j < i : E_j \geq i\}$, where $E_i \geq i$ is the (possibly random but independent of P_i) testing/stopping time (time at which P_i is observed) for hypothesis H_i. Due to Theorem S.2, the FDR-ADDIS-Graph$_{\text{conf}}$ controls the FDR in this setting. We assume that $E_i = i + e$ for some constant test duration $e \in \mathbb{N}_0$. In the following simulations we compare the FDR-ADDIS-Graph$_{\text{conf}}$ and ADDIS$^*_{\text{async}}$ (Tian and Ramdas 2019) in terms of power and FDR for $e \in \{0, 1, 2, 5\}$. Since FDR is less conservative than FWER, we also change the overall level to $\alpha = 0.05$. As recommended (Tian and Ramdas 2019), we choose $\tau_i = 0.5$ and $\lambda_i = 0.25$ for all $i \in \mathbb{N}$, but use the same $(\gamma_i)_{i\in\mathbb{N}}$ as in Section 7. For the weights of the FDR-ADDIS-Graph$_{\text{conf}}$, we choose $g^*_{j,i} = g_{j,i} \Big/ \left(1 - \sum_{k=j+1}^{E_j} g_{j,k}\right)$ if $i > E_j$ and

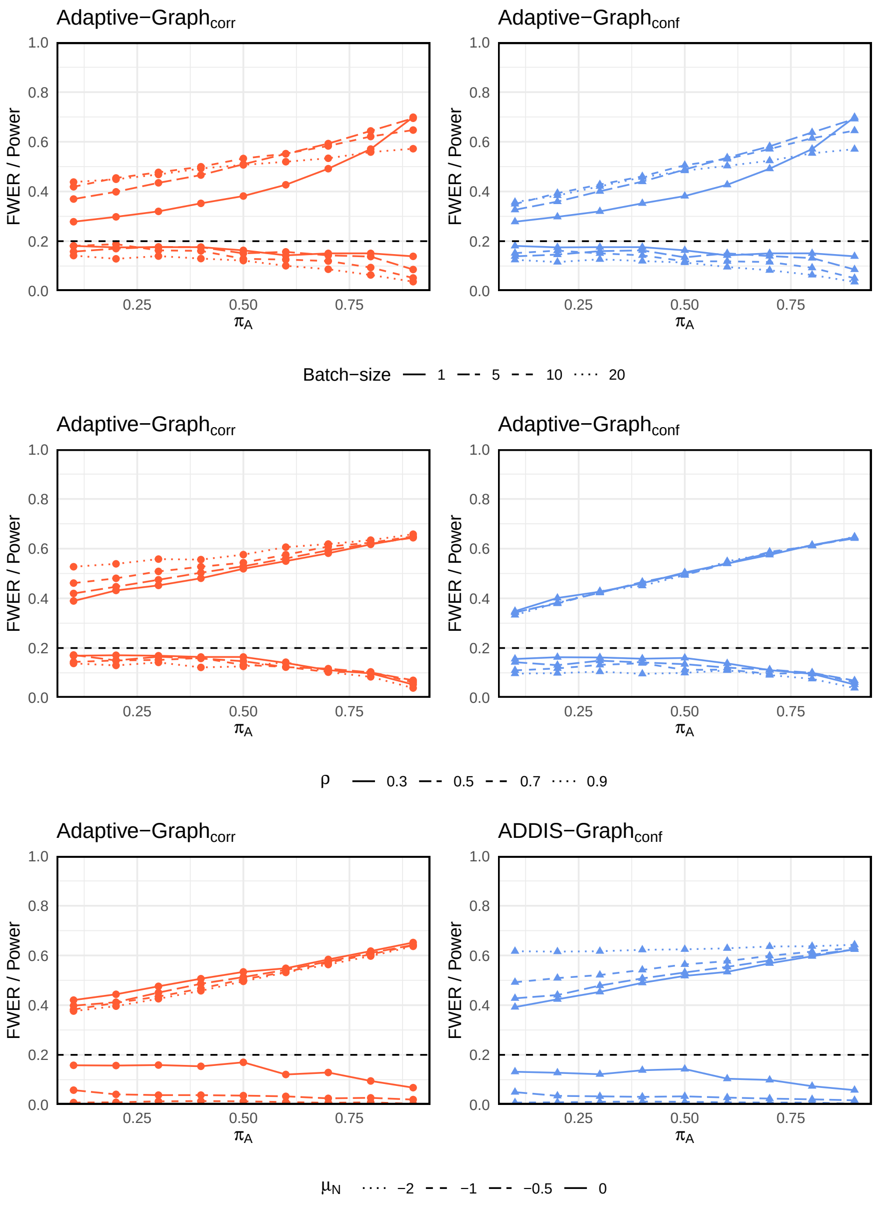

Figure S.5 Comparison of Adaptive-Graph$_{\text{corr}}$ (S.2) and Adaptive-Graph$_{\text{conf}}$ (4) in terms of power and FWER for different proportions of false null hypotheses (π_A). In the top row, we vary the batch-size; In the middle row, we vary the strength of correlation ρ; In the bottom row, we vary the conservativeness of the null p-values μ_N. Lines above the overall level $\alpha = 0.2$ correspond to power and lines below to FWER. The p-values were generated and the procedures applied as described in the text. The Adaptive-Graph$_{\text{corr}}$ allows to increase the power when the batch-size is large and the within-batch correlation is high. However, when the p-values become conservative, the Adaptive-Graph$_{\text{conf}}$ allows to gain power, while the Adaptive-Graph$_{\text{corr}}$ loses a bit of power.

© 2010 WILEY-VCH Verlag GmbH & Co. KGaA, Weinheim

$g^*_{j,i} = 0$ otherwise, where $g_{j,i} = \gamma_{i-j}$ and $h^*_{j,i} = g^*_{j,i}$. Furthermore, we set $W_0 = \alpha$. The results can be found in the Figure S.6.

The results are similar to the results for the FWER controlling procedures (Section 7). The power of ADDIS$^*_{\text{async}}$ decreases enormously for an increasing test duration. This decrease can be decelerated by the FDR-ADDIS-Graph$_{\text{async}}$. Note that the decline in power due to conflict sets is greater than in the FWER case. This is because more significance level can be distributed among the hypotheses in the FDR case, and conflict sets postpone the use of the levels, meaning that a larger portion of the significance level is not used for the first 100 hypotheses. With a larger number of hypotheses or a faster decreasing $(\gamma_i)_{i\in\mathbb{N}}$, the decline due to conflict sets should be smaller.

S.5 Proofs

Proof of Theorem 3.2. Let $(\alpha_i)_{i\in\mathbb{N}}$ be given by the ADDIS-Graph. We need to show that for any $i \in \mathbb{N}$ and $U_{1:i} := (U_1, \dots, U_i)^T \in \{0,1\}^i$:

$$\sum_{j=1}^{i} \frac{\alpha_j}{\tau_j - \lambda_j}(1 - U_j) \leq \alpha. \tag{S.4}$$

Now let $i \in \mathbb{N}$ and $U_{1:i} = (U_1, \dots, U_i)^T \in \{0,1\}^i$ be arbitrary but fixed. With this, (S.4) is equivalent to

$$F_i(U_{1:i}) := \sum_{j=1}^{i} \left(\alpha\gamma_j + \sum_{k=1}^{j-1} g_{k,j} U_k \alpha_k(U_{1:(k-1)}) \frac{1}{\tau_k - \lambda_k} \right) (1 - U_j) \leq \alpha. \tag{S.5}$$

Note that we only wrote the dependence of α_k on $U_{1:(k-1)} = (U_1, \dots, U_{k-1})^T$, although the parameters λ_k and τ_k could depend on it as well. That is, because these parameters could also be fixed, meaning if we change the $U_{1:(k-1)}$ they would still be valid parameters for an ADDIS-Graph. In contrast, the α_k changes by definition. It is difficult to show the validity of (S.5) directly. However, we will see that there exists $\tilde{U}_{1:i} \in \{0,1\}^i$ that obviously fulfil $F_i(\tilde{U}_{1:i}) \leq \alpha$. Therefore, the idea is to determine such a $\tilde{U}_{1:i}$ that additionally satisfies $F_i(U_{1:i}) \leq F_i(\tilde{U}_{1:i})$.

Let $l = \max\{j \in \{1, \dots, i\} : U_j = 1\}$ (we set $\max(\emptyset) = 0$) and $U^l_{1:i} = (U^l_1, \dots, U^l_i)^T$, where $U^l_j = U_j$ for all $j \neq l$ and $U^l_l = 0$. We assume that $l > 0$ (if $l = 0$, we later see $F_i(U_{1:i}) \leq \alpha$ anyway). In the next step we want to show that $F_i(U_{1:i}) \leq F_i(U^l_{1:i})$. For shorter notation we write $\alpha_j = \alpha_j(U_{1:(j-1)})$ and $\alpha^l_j = \alpha_j(U^l_{1:(j-1)})$. Since for all $j \leq i$: $U^l_j = U_j$ ($j \neq l$), $U^l_j = 0$ ($j \geq l$), $U_j = 0$ ($j \geq l+1$) and $\alpha^l_j = \alpha_j$ ($j \leq l$), we have:

© 2010 WILEY-VCH Verlag GmbH & Co. KGaA, Weinheim

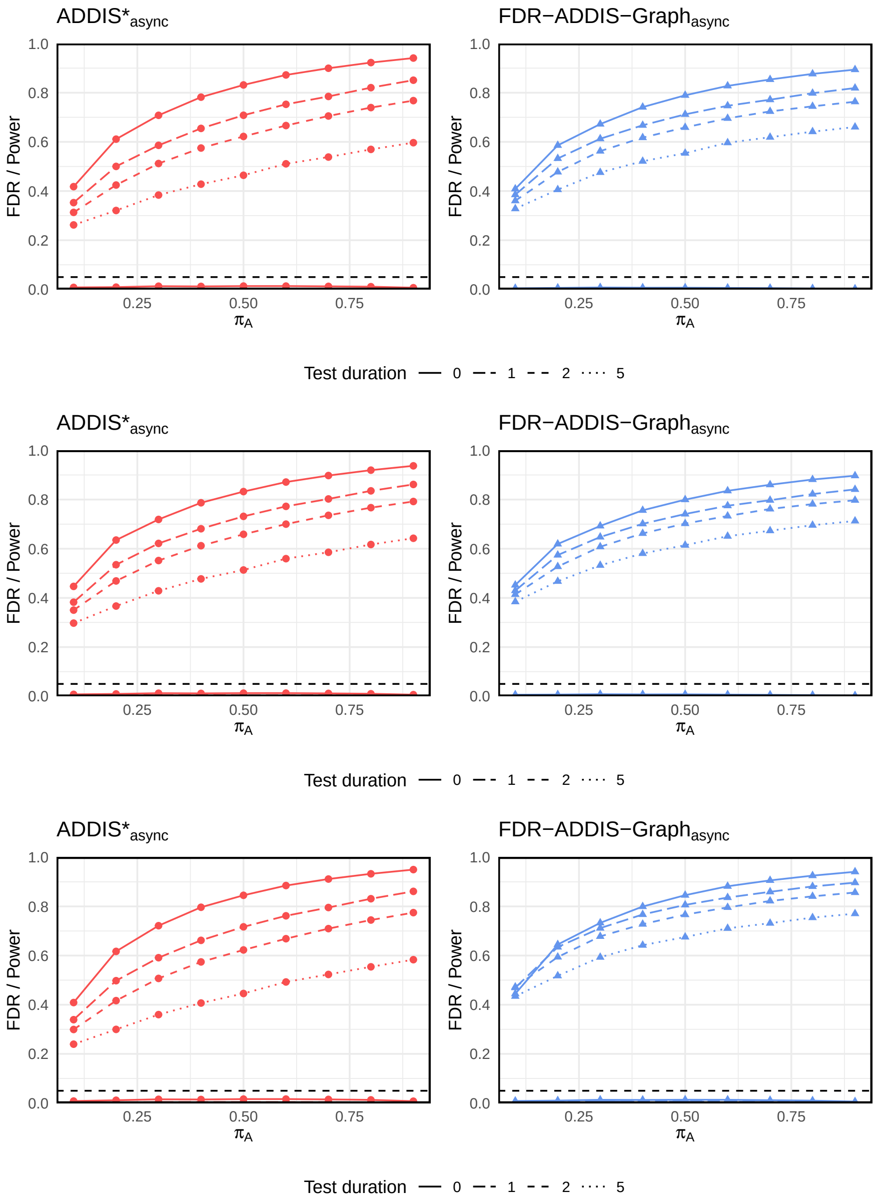

Figure S.6 Comparison of ADDIS$^*_{\text{async}}$ and FDR-ADDIS-Graph$_{\text{conf}}$ in terms of power and FDR for different test durations and proportions of false null hypotheses (π_A). Lines above the overall level $\alpha = 0.05$ correspond to power and lines below to FDR. The p-values were generated as described in the text with parameter $\mu_N = -0.5$. Both procedures were applied with parameters $\tau_i = 0.5$, $\lambda_i = 0.25$ and $W_0 = \alpha$. In the top row $\gamma_i \propto 1/\left((i+1)\log(i+1)^2\right)$, in the middle row $\gamma_i \propto 1/i^{1.6}$ and in the bottom row $\gamma_i = 6/(\pi^2 i^2)$. When hypotheses are not tested asynchronously, the power of both procedures is similar. However, the ADDIS-Spending$_{\text{local}}$ loses power when the test duration increases, while the ADDIS-Graph$_{\text{conf-u}}$ can decelerate this decrease.

© 2010 WILEY-VCH Verlag GmbH & Co. KGaA, Weinheim

$$
\begin{aligned}
&F_i(U^l_{1:i}) - F_i(U_{1:i}) \\
&= \sum_{j=1}^{i} \alpha\gamma_j(1-U^l_j) - \sum_{j=1}^{i} \alpha\gamma_j(1-U_j) + \sum_{j=1}^{i}\left(\sum_{k=1}^{j-1} g_{k,j}U^l_k\alpha^l_k \frac{1}{\tau_k-\lambda_k}\right)(1-U^l_j) \\
&- \sum_{j=1}^{i}\left(\sum_{k=1}^{j-1} g_{k,j}U_k\alpha_k \frac{1}{\tau_k-\lambda_k}\right)(1-U_j) \\
&= \alpha\gamma_l + \sum_{j=1}^{i}\left(\sum_{k=1}^{j-1} g_{k,j}U^l_k\alpha^l_k \frac{1}{\tau_k-\lambda_k}\right)(1-U^l_j) - \sum_{j=1}^{i}\left(\sum_{k=1}^{j-1} g_{k,j}U_k\alpha_k \frac{1}{\tau_k-\lambda_k}\right)(1-U_j) \\
&= \alpha\gamma_l + \sum_{k=1}^{l-1} g_{k,l}U^l_k\alpha^l_k \frac{1}{\tau_k-\lambda_k} + \sum_{j=l+1}^{i}\sum_{k=1}^{l-1} g_{k,j}U^l_k\alpha^l_k \frac{1}{\tau_k-\lambda_k} \\
&- \sum_{j=l+1}^{i}\sum_{k=1}^{l} g_{k,j}U_k\alpha_k \frac{1}{\tau_k-\lambda_k} \\
&= \alpha\gamma_l + \sum_{k=1}^{l-1} g_{k,l}U_k\alpha_k \frac{1}{\tau_k-\lambda_k} - \sum_{j=l+1}^{i} g_{l,j}\alpha_l \frac{1}{\tau_l-\lambda_l} \\
&\geq \alpha\gamma_l + \sum_{k=1}^{l-1} g_{k,l}U_k\alpha_k \frac{1}{\tau_k-\lambda_k} - \alpha_l \frac{1}{\tau_l-\lambda_l} \overset{Def.1}{=} 0,
\end{aligned}
$$

where we used in the inequality that the sequence $(g_{l,j})_{j=l+1}^{\infty}$ is non-negative and sums to at most 1 for all $l \in \mathbb{N}$.

Since the $U_{1:i} \in \{0,1\}^i$ was arbitrary, this shows $F_i(U_{1:i}) \leq F_i(U^0_{1:i})$ for all $U_{1:i} \in \{0,1\}^i$, where $U^0_{1:i} = (0,\dots,0)^T \in \{0,1\}^i$. Next, we deduce that $F_i(U^0_{1:i}) \leq \alpha$ and conclude the proof. For this, just recognize that $U^0_{1:i}$ means $U_j = 0$ for all $j \leq i$. Hence, we obtain

$$F_i(U^0_{1:i}) = \sum_{j=1}^{i} \alpha\gamma_j \leq \alpha.$$

□

Proof of Proposition 3.3. Every online procedure satisfying equation (1) is a sequence of non-negative random variables $(\alpha_i)_{i\in\mathbb{N}}$, where α_i is measurable with respect to $\mathcal{G}_{i-1}$, such that

$$\sum_{j\leq i} \frac{\alpha_j}{\tau_j-\lambda_j}(1-U_j) \leq \alpha \quad \text{for all } i \in \mathbb{N}. \tag{S.6}$$

Note that α_i is fully determined through $P_1,\dots,P_{i-1}$. Hence, pessimistic assumptions about U_i need to be made at step $i \in \mathbb{N}$ in order to satisfy equation (S.6). Consequently, condition (1) is equivalent to

$$0 \leq \alpha_i \leq (\tau_i-\lambda_i)\left(\alpha - \sum_{j\leq i-1} \frac{\alpha_j}{\tau_j-\lambda_j}(1-U_j)\right) \quad \text{for all } i \in \mathbb{N}. \tag{S.7}$$

© 2010 WILEY-VCH Verlag GmbH & Co. KGaA, Weinheim

Let $i \in \mathbb{N}$ be arbitrary but fixed. In addition, let $(\alpha_j)_{j<i}$ be levels obtained by an ADDIS-Graph with parameters $(\gamma_j)_{j<i}$ and $(g_{k,j})_{k<j<i}$. We want to prove that

$$\alpha_i(\gamma_i, (g_{j,i})_{j<i})) = (\tau_i - \lambda_i)\left(\alpha\gamma_i + \sum_{j=1}^{i-1} g_{j,i} U_j \frac{\alpha_j}{\tau_i - \lambda_j}\right),$$

where $\gamma_i \in \left[0, 1 - \sum_{j=1}^{i-1}\gamma_j\right]$ and $g_{j,i} \in \left[0, 1 - \sum_{k=j+1}^{i-1} g_{j,k}\right]$, $j \in \{1, \ldots, i-1\}$, can take any value in the interval $\left[0, (\tau_i - \lambda_i)\left(\alpha - \sum_{j \le i-1} \frac{\alpha_j}{\tau_j - \lambda_j}(1 - U_j)\right)\right]$. Since α_i is continuous in γ_i and $(g_{j,i})_{j<i}$, it is sufficient to show that $\alpha_i(0, (0)_{j<i}) = 0$ and $\alpha_i\left(1 - \sum_{j=1}^{i-1}\gamma_j, \left(1 - \sum_{k=j+1}^{i-1} g_{j,k}\right)_{j<i}\right) = (\tau_i - \lambda_i)\left(\alpha - \sum_{j \le i-1} \frac{\alpha_j}{\tau_j - \lambda_j}(1 - U_j)\right)$. The first equation follows immediately, hence we only need to show the second:

$$\begin{aligned}
&\alpha_i\left(1 - \sum_{j=1}^{i-1}\gamma_j, \left(1 - \sum_{k=j+1}^{i-1} g_{j,k}\right)_{j<i}\right) - (\tau_i - \lambda_i)\left(\alpha - \sum_{j=1}^{i-1}\frac{\alpha_j}{\tau_j - \lambda_j}(1 - U_j)\right) \\
&= (\tau_i - \lambda_i)\left(\alpha\left(1 - \sum_{j=1}^{i-1}\gamma_j\right) + \sum_{j=1}^{i-1}\left(1 - \sum_{k=j+1}^{i-1} g_{j,k}\right) U_j \frac{\alpha_j}{\tau_i - \lambda_j}\right) \\
&- (\tau_i - \lambda_i)\left(\alpha - \sum_{j=1}^{i-1}\left(\alpha\gamma_j + \sum_{k=1}^{j-1} g_{k,j} U_k \frac{\alpha_k}{\tau_k - \lambda_k}\right)(1 - U_j)\right) \\
&= (\tau_i - \lambda_i)\left(-\alpha\sum_{j=1}^{i-1}\gamma_j U_j + \sum_{j=1}^{i-1} U_j \frac{\alpha_j}{\tau_i - \lambda_j} - \sum_{j=1}^{i-1}\sum_{k=j+1}^{i-1} g_{j,k} U_j \frac{\alpha_j}{\tau_i - \lambda_j}\right. \\
&\left. + \sum_{j=1}^{i-1}\sum_{k=1}^{j-1} g_{k,j} U_k \frac{\alpha_k}{\tau_k - \lambda_k} - \sum_{j=1}^{i-1}\left(\sum_{k=1}^{j-1} g_{k,j} U_k \frac{\alpha_k}{\tau_k - \lambda_k}\right) U_j\right) \\
&= (\tau_i - \lambda_i)\left(\sum_{j=1}^{i-1} U_j \frac{\alpha_j}{\tau_i - \lambda_j} - \sum_{j=1}^{i-1} U_j\left(\alpha\gamma_j + \sum_{k=1}^{j-1} g_{k,j} U_k \frac{\alpha_k}{\tau_k - \lambda_k}\right)\right) = 0.
\end{aligned}$$

Therefore, if some online Procedure $(\tilde{\alpha}_i)_{i\in\mathbb{N}}$ satisfying condition (1) is given, we can choose the parameters $(\gamma_i)_{i\in\mathbb{N}}$ and $(g_{j,i})_{j\in\mathbb{N}, i>j}$ such that for the individual significance levels of the ADDIS-Graph $(\alpha_i)_{i\in\mathbb{N}}$ holds $\alpha_i = \tilde{\alpha}_i$ for all $i \in \mathbb{N}$. □

Proof of Lemma 5.1. In the following we write $\tilde{\alpha}_i^{\text{ind}} = \frac{\alpha_i^{\text{ind}}}{\tau_i - \lambda_i}$ and $\tilde{\alpha}_i^{\text{loc}} = \frac{\alpha_i^{\text{loc}}}{\tau_i - \lambda_i}$. Let $g_{j,i} = \frac{\gamma_{t(j)+i-j-1} - \gamma_{t(j)+i-j}}{\gamma_{t(j)}}$, $i > j$, where $t(j) = 1 + \sum_{k<j}(1 - U_k)$. Obviously, $\tilde{\alpha}_1^{\text{ind}} = \alpha\gamma_{t(1)}$. Now assume $\tilde{\alpha}_j^{\text{ind}} = \alpha\gamma_{t(j)}$ for all $j < i$. Thus, we have

$$\begin{aligned}
\tilde{\alpha}_i^{\text{ind}} &= \alpha\gamma_i + \alpha\sum_{j=1}^{i-1} U_j(\gamma_{i-j+\sum_{k<j}(1-U_k)} - \gamma_{i-j+1+\sum_{k<j}(1-U_k)}) \\
&= \alpha\gamma_i + \alpha\sum_{j=t(i)}^{i-1}(\gamma_j - \gamma_{j+1}) = \alpha\gamma_{t(i)}.
\end{aligned}$$

© 2010 WILEY-VCH Verlag GmbH & Co. KGaA, Weinheim

With this, we can show that the ADDIS-Spending$_{\text{local}}$ can obtained by α_i^{loc} with the same choice of weights

$$\begin{aligned}\tilde{\alpha}_i^{\text{loc}} &= \alpha\gamma_i + \alpha \sum_{j=1}^{i-L_i-1} U_j(\gamma_{i-j+\sum_{k<j}(1-U_k)} - \gamma_{i-j+1+\sum_{k<j}(1-U_k)}) \\ &= \alpha\gamma_i + \alpha \sum_{j=t(i)^{\text{loc}}}^{i-1} (\gamma_j - \gamma_{j+1}) = \alpha\gamma_{t(i)^{\text{loc}}},\end{aligned}$$

where $t(i)^{\text{loc}} = 1 + L_i + \sum_{j=1}^{i-L_i-1}(1-U_j)$. □

Proof of Proposition 5.2. Let $g_{j,i} = \frac{\gamma_{t(j)+i-j-1} - \gamma_{t(j)+i-j}}{\gamma_{t(j)}}$, $i > j$, where $t(j) = 1 + \sum_{k<j}(1-U_k)$ and $(g^*_{j,i})_{j\in\mathbb{N},i>j}$ be defined as in Algorithm S.1.

Algorithm S.1 Local dependence adjusted weights for uniform improvement

$g^*_{j,i} \leftarrow g_{j,i}\ \forall j \in \mathbb{N}, i > j$
for $j = 1, 2, \dots$ **do**
  for $i = j+1, j+2, \dots$ **do**
    if $i - L_i \leq j$ **then**
      $g^-_{j,i} \leftarrow g^*_{j,i}$
      $g^*_{j,i} \leftarrow 0$
      for $k > i$ **do** $g^*_{j,k} \leftarrow g^*_{j,k} + g^-_{j,i} g_{i,k}$
      end for
    else
      $g^-_{j,i} \leftarrow \sum_{l=i-L_i}^{i-1} g_{l,i} g^-_{j,l}$
      $g^*_{j,i} \leftarrow g^*_{j,i} - g^-_{j,l}$
      for $k > i$ **do** $g^*_{j,k} \leftarrow g^*_{j,k} + g^-_{j,i} g_{i,k}$
      end for
    end if
  end for
end for

The weight $g^-_{j,i}$ defined in Algorithm S.1 can be interpreted as the part of $g^*_{j,i}$ that cannot be used at step i due to local dependence and thus is distributed to the future weights $g^*_{j,k}$, $k > i$, according to the weights $g_{i,k}$. In case of $i - L_i \leq j$, H_i is not allowed to use any significance level of H_j, which is why we set $g^-_{j,i} = g^*_{j,i}$ and thus $g^*_{j,i} = 0$. This ensures that $g^*_{j,i} = 0$ for all $j \in \mathcal{X}_i$, as required in Definition 4.1. Setting $g^-_{j,i} = \sum_{l=i-L_i}^{i-1} g_{l,i} g^-_{j,l}$ in case of $i - L_i > j$ additionally ensures that $g^*_{j,i}$ solely depends on $(g_{l,k})_{l\leq i-L_i,k>l}$, which is measurable with respect to $\sigma(P_1, \dots, P_{i-L_i-1}) = \mathcal{G}_{-\mathcal{X}_i}$. Furthermore, note that the sum of the $(g^*_{j,i})_{i\geq j+1}$ is less or equal than the sum of $(g_{j,i})_{i\geq j+1}$ for each $j \in \mathbb{N}$ and since

$$\sum_{i=j+1}^{\infty} g_{j,i} = \frac{1}{\gamma_{t(j)}} \sum_{i=j+1}^{\infty} \gamma_{t(j)+i-j-1} - \gamma_{t(j)+i-j} = 1,$$

$(g^*_{j,i})_{j\in\mathbb{N},i>j}$ can be used in the ADDIS-Graph$_{\text{conf}}$ (Definition 4.1). In the following, we show that the ADDIS-Graph$_{\text{conf}}$ with this choice of $(g^*_{j,i})_{j\in\mathbb{N},i>j}$ leads to a uniform improvement over ADDIS-Spending$_{\text{local}}$.

We need to show that $\tilde{\alpha}_i^{\text{loc}}$, defined in the proof of Lemma 5.1, is less or equal than $\tilde{\alpha}_i = \alpha\gamma_i + \sum_{j=1}^{i-L_i-1} g^*_{j,i} U_j \alpha_j$ for all $i \in \mathbb{N}$. For this, we define $g^{+,\text{loc}}_{j,i}$ and $g^+_{j,i}$ as the proportion of $\alpha\gamma_j$ that is shifted to $\tilde{\alpha}_i^{\text{loc}}$ and $\tilde{\alpha}_i$, respectively, in case of $P_j \leq \lambda_j$ or $P_j > \tau_j$. Hence, it is sufficient to show that $g^{+,\text{loc}}_{j,i} \leq g^+_{j,i}$

© 2010 WILEY-VCH Verlag GmbH & Co. KGaA, Weinheim

Algorithm S.2 Calculation of $g^{+,\text{loc}}_{j,i}$ for $j \in \mathbb{N}$

$g^{+,\text{loc}}_{j,i} \leftarrow g_{j,i}\ \forall i > j$
for $i = j + 1, j + 2, \ldots$ **do**
  if $i - L_i \leq j$ **then**
    $g^{-}_{j,i} \leftarrow g^{+,\text{loc}}_{j,i}$
    $g^{+,\text{loc}}_{j,i} \leftarrow 0$
    for $k > i$ **do** $g^{+,\text{loc}}_{j,k} \leftarrow g^{+,\text{loc}}_{j,k} + g^{-}_{j,i} g_{i,k} U_i$
    end for
  else
    $g^{-}_{j,i} \leftarrow \sum_{l=i-L_i}^{i-1} g_{l,i} g^{-}_{j,l} U_l + \sum_{l=i-L_i}^{i-1} g_{l,i} g^{+}_{j,l} U_l$
    $g^{+,\text{loc}}_{j,i} \leftarrow g^{+,\text{loc}}_{j,i} - g^{-}_{j,l}$
    for $k > i$ **do** $g^{+,\text{loc}}_{j,k} \leftarrow g^{+,\text{loc}}_{j,k} + g^{-}_{j,i} g_{i,k} U_i + g^{+}_{j,i} g_{i,k} U_i$
    end for
  end if
end for

Algorithm S.3 Calculation of $g^{+}_{j,i}$ for $j \in \mathbb{N}$

$g^{+}_{j,i} \leftarrow g_{j,i}\ \forall i > j$
for $i = j + 1, j + 2, \ldots$ **do**
  if $i < d_j$ **then**
    $g^{-}_{j,i} \leftarrow g^{+}_{j,i}$
    $g^{+}_{j,i} \leftarrow 0$
    for $k > i$ **do** $g^{+}_{j,k} \leftarrow g^{+}_{j,k} + g^{-}_{j,i} g_{i,k}$
    end for
  else
    $g^{-}_{j,i} \leftarrow \sum_{l=i-L_i}^{i-1} g_{l,i} g^{-}_{j,l} + \sum_{l=i-L_i}^{i-1} g_{l,i} g^{+}_{j,l} U_l$
    $g^{+}_{j,i} \leftarrow g^{+}_{j,i} - g^{-}_{j,l}$
    for $k > i$ **do** $g^{+}_{j,k} \leftarrow g^{+}_{j,k} + g^{-}_{j,i} g_{i,k} + g^{+}_{j,i} g_{i,k} U_i$
    end for
  end if
end for

for all $j \in \mathbb{N}$ and $i > j$. In Algorithms S.2 and S.3 we show how $g^{+,\text{loc}}_{j,i}$ and $g^{+}_{j,i}$, respectively, can be calculated. Since $U_j \leq 1$, we have $g^{+}_{j,i} \geq g^{\text{spend},+}_{j,i}$ and the assertion follows.

□

© 2010 WILEY-VCH Verlag GmbH & Co. KGaA, Weinheim

Proof of Theorem S.1. First, note that

$$\begin{aligned}
\text{FWER}(i) &= \left(\bigcup_{j \le i, j \in I_0} \{P_i \le \alpha_i\} \right) \\
&\le \sum_{j \le i, j \in I_0} \mathbb{P} \left(\bigcap_{k \in B_{b_j}, k < j, k \in I_0} \{P_k > \alpha_k\} \cap \{P_j \le \alpha_j\} \right) \\
&\le \sum_{j \le i, j \in I_0} \mathbb{P} \left(\bigcap_{k \in B_{b_j}, k < j, k \in I_0, C_k = 0} \{P_k > \alpha_k\} \cap \{P_j \le \alpha_j\} \right) \\
&= \sum_{j \le i, j \in I_0} \mathbb{E} \left[\mathbb{P} \left(\bigcap_{k \in B_{b_j}, k < j, k \in I_0, C_k = 0} \{P_k > \alpha_k\} \cap \{P_j \le \alpha_j\} \middle| \mathcal{F}_{b_j - 1} \right) \right] \\
&= \sum_{j \le i, j \in I_0} \mathbb{E} \left[\alpha_j^{c, I_0} \right] \\
&\le \sum_{j \le i, j \in I_0} \mathbb{E} \left[\alpha_j^{c, I_0} \mathbb{E} \left(\frac{1 - C_j}{1 - \lambda_{b_j}} \middle| \mathcal{F}_{b_j - 1} \right) \right] \\
&= \mathbb{E} \left[\sum_{j \le i, j \in I_0} \alpha_j^{c, I_0} \frac{1 - C_j}{1 - \lambda_{b_j}} \right],
\end{aligned}$$

where $\alpha_j^{c,I_0} = \mathbb{P} \left(\bigcap_{k \in B_{b_j}, k < j, k \in I_0, C_k = 0} \{P_k > \alpha_k\} \cap \{P_j \le \alpha_j\} \middle| \mathcal{F}_{b_j - 1} \right)$. Using the subset pivotality condition, we obtain

$$\begin{aligned}
\sum_{j \le i, j \in I_0} \alpha_j^{c, I_0} \frac{1 - C_j}{1 - \lambda_{b_j}} &= \sum_{j=1}^{b_i} \frac{1}{1 - \lambda_{b_j}} \mathbb{P} \left(\bigcup_{k \in B_{b_j}, k \le i, k \in I_0, C_k = 0} \{P_k \le \alpha_k\} \middle| \mathcal{F}_{b_j - 1} \right) \\
&\le \sum_{j=1}^{b_i} \frac{1}{1 - \lambda_{b_j}} \mathbb{P}_{H_{\mathbb{N}}} \left(\bigcup_{k \in B_{b_j}, k \le i, C_k = 0} \{P_k \le \alpha_k\} \middle| \mathcal{F}_{b_j - 1} \right) \\
&= \sum_{j=1}^{i} \frac{1}{1 - \lambda_{b_j}} \alpha_j^c (1 - C_j) \le \alpha.
\end{aligned}$$

□

Proof of Theorem S.4. Obviously, α_i is measurable regarding $\mathcal{F}_{b_i - 1}$. Now let $i \in \mathbb{N}$ be fix. We define

$$A(k) := \sum_{j=1}^{i-k} \frac{\alpha_j^c + (\alpha_j - \alpha_j^c) \sum_{l=i-k+1}^{i} g_{j,l}^*}{1 - \lambda_{b_j}} (1 - C_j) + \frac{\alpha_j \sum_{l=i-k+1}^{i} g_{j,l}^*}{1 - \lambda_{b_j}} C_j.$$

© 2010 WILEY-VCH Verlag GmbH & Co. KGaA, Weinheim

For $k \in \{0, \ldots, i-1\}$, we can write

$$
\begin{aligned}
A(k) &= \sum_{j=1}^{i-k} \frac{\alpha_j^c + (\alpha_j - \alpha_j^c)\sum_{l=i-k+1}^{i} g_{j,l}^*}{1-\lambda_{b_j}}(1-C_j) + \frac{\alpha_j \sum_{l=i-k+1}^{i} g_{j,l}^*}{1-\lambda_{b_j}} C_j \\
&\leq \frac{\alpha_{i-k}}{1-\lambda_{b_{i-k}}} + \sum_{j=1}^{i-k-1} \frac{\alpha_j^c + (\alpha_j - \alpha_j^c)\sum_{l=i-k+1}^{i} g_{j,l}^*}{1-\lambda_{b_j}}(1-C_j) + \frac{\alpha_j \sum_{l=i-k+1}^{i} g_{j,l}^*}{1-\lambda_{b_j}} C_j \\
&= \alpha\gamma_{i-k} + A(k+1).
\end{aligned}
$$

Since $A(i) = 0$, we especially have

$$
A(0) = \sum_{j=1}^{i} \frac{\alpha_j^c}{1-\lambda_{b_j}}(1-C_j) \leq \alpha \sum_{j=1}^{i} \gamma_j \leq \alpha.
$$

□

Proof of Theorem S.2. Let $\alpha_j^0 = W_0\gamma_j + \sum_{k=1}^{j-1} h_{k,j}^* R_k[\alpha K_k + (\alpha - W_0)K_k^c]$ for all $j \in \mathbb{N}$. Note that

$$
\begin{aligned}
\sum_{j=1}^{i} \alpha_j^0 &= \sum_{j=1}^{i} W_0\gamma_j + \sum_{j=1}^{i}\sum_{k=1}^{j-1} h_{k,j}^* R_k[\alpha K_k + (\alpha - W_0)K_k^c] \\
&\leq W_0 + \sum_{k=1}^{i-1} R_k[\alpha K_k + (\alpha - W_0)K_k^c] \sum_{j=k+1}^{i} h_{k,j}^* \\
&\leq W_0 + \sum_{k=1}^{i-1} R_k[\alpha K_k + (\alpha - W_0)K_k^c] \\
&= W_0 + (\alpha - W_0)K_i + \alpha(|R(i-1)| - 1)K_i \\
&\leq \alpha(|R(i)| \vee 1)
\end{aligned}
$$

With this, it can be shown that the ADDIS-Graph$_{\text{conf}}$ satisfies (1) in the same way as in Theorem 3.2 by replacing $\alpha\gamma_j$ with α_j^0 on the left side in equation (S.5) and α with $\alpha(|R(i)| \vee 1)$ on the right side. Hence, if the null p-values are independent from each other and the non-nulls and α_i, λ_i and τ_i are monotonic functions of the past, then the FDR control follows immediately by Theorem 1 of Tian and Ramdas (2019). Furthermore, the mFDR control for $\tau_i = 1$, $i \in \mathbb{N}$, follows by Theorem 2 of Zrnic et al. (2021). However, since we consider general $\tau_i \in (0, 1]$, we provide a self-contained proof for mFDR control when (S.3) is fulfilled, which is very similar to the proofs Zrnic et al. (2021) and Tian and Ramdas (2019).

© 2010 WILEY-VCH Verlag GmbH & Co. KGaA, Weinheim

Consider

$$
\begin{aligned}
\mathbb{E}[|V(i)|] &= \sum_{j\in I_0, j\leq i} \mathbb{E}[R_j] \\
&= \sum_{j\in I_0, j\leq i} \mathbb{E}[\mathbb{P}(P_j \leq \alpha_j | P_j \leq \tau_j, \mathcal{F}_{-\mathcal{X}_j})\mathbb{P}(P_j \leq \tau_j | \mathcal{F}_{-\mathcal{X}_j})] \\
&\leq \sum_{j\in I_0, j\leq i} \mathbb{E}\left[\frac{\alpha_j}{\tau_j}\mathbb{P}(P_j \leq \tau_j | \mathcal{F}_{-\mathcal{X}_j})\right] \\
&\leq \sum_{j\in I_0, j\leq i} \mathbb{E}\left[\frac{\alpha_j}{\tau_j}\mathbb{P}(P_j \leq \tau_j | \mathcal{F}_{-\mathcal{X}_j})\frac{\mathbb{P}(P_j > \lambda_j | P_j \leq \tau_j, \mathcal{F}_{-\mathcal{X}_j})}{1-\lambda_j/\tau_j}\right] \\
&= \sum_{j\in I_0, j\leq i} \mathbb{E}\left[\frac{\alpha_j}{\tau_j - \lambda_j}\mathbb{1}\{\lambda_j < P_j \leq \tau_j\}\right] \\
&\leq \mathbb{E}[|R(i)| \vee 1]\alpha,
\end{aligned}
$$

where the first and second inequality follow from the uniform validity of the null p-values and the third inequality from condition (S.3).

□

References

Bretz, F., Posch, M., Glimm, E., Klinglmueller, F., Maurer, W., and Rohmeyer, K. (2011). Graphical approaches for multiple comparison procedures using weighted bonferroni, simes, or parametric tests. *Biometrical Journal*, 53(6):894–913.

Feng, J., Emerson, S., and Simon, N. (2021). Approval policies for modifications to machine learning-based software as a medical device: A study of bio-creep. *Biometrics*, 77(1):31–44.

Feng, J., Pennllo, G., Petrick, N., Sahiner, B., Pirracchio, R., and Gossmann, A. (2022). Sequential algorithmic modification with test data reuse. In *Uncertainty in Artificial Intelligence*, pages 674–684. PMLR.

Fischer, L., Bofill Roig, M., and Brannath, W. (2024). The online closure principle. *The Annals of Statistics*, 52(2):817–841.

Robertson, D. S., Wason, J. M., König, F., Posch, M., and Jaki, T. (2023). Online error rate control for platform trials. *Statistics in Medicine*.

Tian, J. and Ramdas, A. (2019). ADDIS: an adaptive discarding algorithm for online FDR control with conservative nulls. *Advances in neural information processing systems*, 32.

Tian, J. and Ramdas, A. (2021). Online control of the familywise error rate. *Statistical Methods in Medical Research*, 30(4):976–993.

Westfall, P. H. and Young, S. S. (1993). *Resampling-based multiple testing: Examples and methods for p-value adjustment*, volume 279. John Wiley & Sons.

Zrnic, T., Ramdas, A., and Jordan, M. I. (2021). Asynchronous online testing of multiple hypotheses. *Journal of Machine Learning Research*, 22:33–1.

© 2010 WILEY-VCH Verlag GmbH & Co. KGaA, Weinheim

© 2010 WILEY-VCH Verlag GmbH & Co. KGaA, Weinheim
