# Supplementary material for: ADDIS‐Graphs for Online Error Control With Application to Platform Trials
Source: Biom J. 2025 Sep 28;67(5):e70075. doi: 10.1002/bimj.70075 (PMC12476829; doi:10.1002/bimj.70075)
Supplement: Supplementary file 2 — Supporting File 2: bimj70075‐sup‐0002‐DataCode.zip. [file BIMJ-67-e70075-s001.zip › Adaptive-Discard-Graph-main/results/Figure7.pdf]

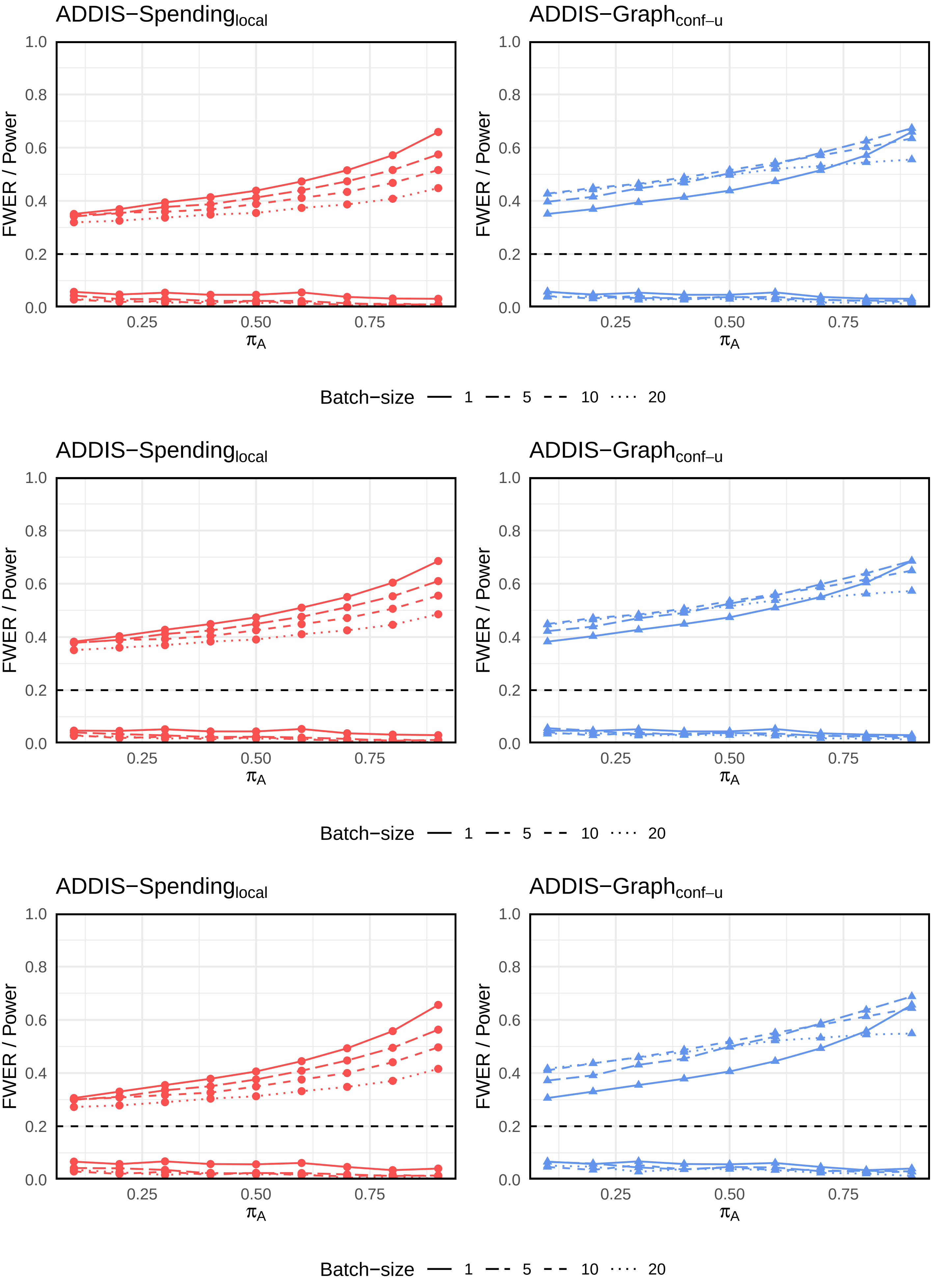

ADDIS−Spending$_{local}$
ADDIS−Graph$_{conf-u}$
FWER / Power
1.0
0.8
0.6
0.4
0.2
0.0
0.25
0.50
0.75
π_A
Batch−size
1
5
10
20
